# Exercise promotes host anti-infection activity through CNDP2 metabolite-mediated inflammasome hyperactivation

**DOI:** 10.1371/journal.ppat.1014554

**Published:** 2026-10-09

**Authors:** Yan Qian, Xiangyun Cheng, Qiannv Liu, Chunlei Wang, Chun Kong, Mengqian Li, Xiangyang Geng, Liya Ai, Mindi Zhao, Shuo Wang, Dong Jiang, Pengyan Xia

**Affiliations:** 1 Beijing Life Science Academy, Beijing, China; 2 Department of Immunology, School of Basic Medical Sciences, State Key Laboratory of Molecular Oncology, Peking University, Beijing, China; 3 Key Laboratory of Molecular Immunology, Chinese Academy of Medical Sciences, Beijing, China; 4 Department of Immunology, School of Basic Medical Sciences, Peking University, Beijing, China; 5 Department of Sports Medicine, Peking University Third Hospital, Beijing, China; 6 Beijing Key Laboratory of Sports Injuries, Institute of Sports Medicine of Peking University, Beijing, China; 7 Department of Laboratory Medicine, Beijing Hospital, National Center for Gerontology, National Clinical Research Center for Gerontology, The Key Laboratory of Geriatrics of NHC, Institute of Geriatric Medicine, Chinese Academy of Medical Sciences, Beijing, China; 8 CAS Key Laboratory of Pathogen Microbiology and Immunology, Institute of Microbiology, Chinese Academy of Sciences, Beijing, China; University of Florida, UNITED STATES OF AMERICA

## Abstract

Exercise can enhance the body’s ability to resist infections, but its molecular mechanisms are still unclear. Here, we found that exercise can induce a hyperactivation-like state of the NLRP3 inflammasome during infection, which enhances the body’s ability to resist infection by continuously secreting inflammatory cytokines and reducing immune cell pyroptosis. At the molecular level, exercise enhances the binding between CNDP2 and GSDMD, promoting the production of γ-Glu-Cys. γ-Glu-Cys binds to CypD and inhibits the binding between CypD and GSDMD, thereby avoiding further damage to the mitochondrial inner membrane during the occurrence of inflammasomes. Limited mitochondrial DNA leakage allows the NLRP3 inflammasome to a hyperactivate state. Our findings reveal the molecular mechanisms by which exercise enhances the body’s ability to resist infections, and provide possible standards for better guiding exercise intensity to improve anti-infection capabilities in the future.

## Author summary

Exercise triggers the production of numerous metabolites. Here, we discovered that exercise promotes the binding between CNDP2 and GSDMD in macrophages, leading to the generation of γ-Glu-Cys. During inflammasome activation, γ-Glu-Cys binds to mitochondrial CypD and prevents it from interacting with the GSDMD N-terminus. This restricts excessive permeabilization of the mitochondrial inner membrane, resulting in a controlled, limited release of mitochondrial DNA. Rather than suppressing the inflammasome, this controlled mitochondrial signal shifts the response into a hyperactivation-like state, characterized by sustained IL-1β secretion without rapid cell lysis. Consequently, exercise-trained macrophages exhibit prolonged and enhanced anti-infection activity. Our work identifies γ-Glu-Cys as an exercise-induced metabolite, providing a mechanistic explanation for why moderate exercise is beneficial for host defense against infections. These insights may open new avenues for developing immunomodulatory strategies that mimic the beneficial effects of exercise.

## Introduction

NOD-, LRR- and pyrin domain-containing protein 3 (NLRP3) inflammasome plays a crucial role in regulating immune responses to various exogenous and endogenous stimuli [1–4]. Inflammasome activation leads to auto-cleavage of caspase-1 and subsequent cleavage of gasdermin D (GSDMD), promoting the release of interleukin-1β (IL-1β) and IL-18, and pyroptosis, a type of cell death caused by GSDMD pores on cell membranes [5–8]. Mitochondria are closely related to the activation of inflammasomes and can activate NLRP3 through pathways such as reactive oxygen species and mitochondrial DNA release, exacerbating the inflammatory responses [9–11]. In addition, the cleaved GSDMD NH2-terminus (N-terminus) can also form pores on the mitochondrial membrane, further promoting cell pyroptosis [12,13]. Controlling the activation intensity of NLRP3 inflammasome is of great significance for regulating inflammatory response and treating related diseases. The hyperactivation state of NLRP3 inflammasome is a unique state in which IL-1β is continuously released while cell pyroptosis is inhibited [14,15]. The specific mechanism of this activation state and the role of mitochondria in this process are not fully unraveled.

The relationship between exercise and anti-infection activity has always been a hot topic in the field of immunology, and an increasing number of studies have shown that moderate exercise can enhance immune function and regulate inflammatory responses [16,17]. Exercise can stimulate the catalytic synthesis of N-lactoyl-phenylalanine (Lac-Phe) by carnosine dipeptidase II (CNDP2), which plays a key role in regulating energy metabolism and immune responses [18–22]. CNDP2 is a type of cytoplasmic non-specific dipeptidase that is expressed in various cells such as immune cells and epithelial cells [23]. Another hydrolytic substrate of CNDP2 is the cysteine-glycine (Cys-Gly) dipeptide, suggesting the substrate selectivity of CNDP2 is flexible [24]. Gamma-glutamylcysteine (γ-Glu-Cys) is an intermediate dipeptide in the glutathione (GSH) synthesis pathway, which serves as a precursor of GSH [25,26]. γ-Glu-Cys also has anti-inflammatory activity, which can inhibit LPS induced accumulation of reactive oxygen species and GSH consumption, demonstrating therapeutic potential for sepsis [27]. In-depth exploration of the catalytic activity of CNDP2 and its products in the anti-infection activity during exercise is of great significance.

Here we show that the hyperactivation of NLRP3 inflammasome is a key factor in enhancing the body’s anti-infection ability during exercise. Exercise promotes the binding of CNDP2 and GSDMD, and facilitates the conversion of GSH into γ-Glu-Cys. γ-Glu-Cys inhibits the binding of CypD to GSDMD, thereby preventing excessive permeabilization of the mitochondrial inner membrane during infection and leading to the occurrence of inflammasome hyperactivation with limited pyroptosis. Our findings reveal the molecular mechanism of exercise in combating infections, providing possibilities for better guiding exercise to enhance the body’s ability to resist infections in the future.

## Results

### Exercise promotes body’s anti-infection activity

Exercise can improve the body’s ability to resist infections, but its mechanism is not fully understood. We established a mouse model of infection post exercise. After a month of continuous and regular exercise, mice were infected with *Klebsiella pneumoniae*, a common gram-negative bacterium that causes pneumonia, an acute respiratory infection in clinical. We found that mice underwent exercise showed better resistance to *K. pneumoniae* compared to mice that did not exercise (Fig 1A–1F). Specifically, there was more serum IL-1β in exercised mouse than sedentary ones (Fig 1A), while there was little change of serum IL-6 or TNF-α between these two groups (Fig 1B and 1C). There was more lymphocyte infiltration in exercised mouse lung tissues post infection (Fig 1D and 1E). The survival rate of exercised mice significantly increased post lethal dose infection (Fig 1F). These results indicate that continuous and regular exercise can indeed improve the host’s resistance to infections.

**Fig 1:**
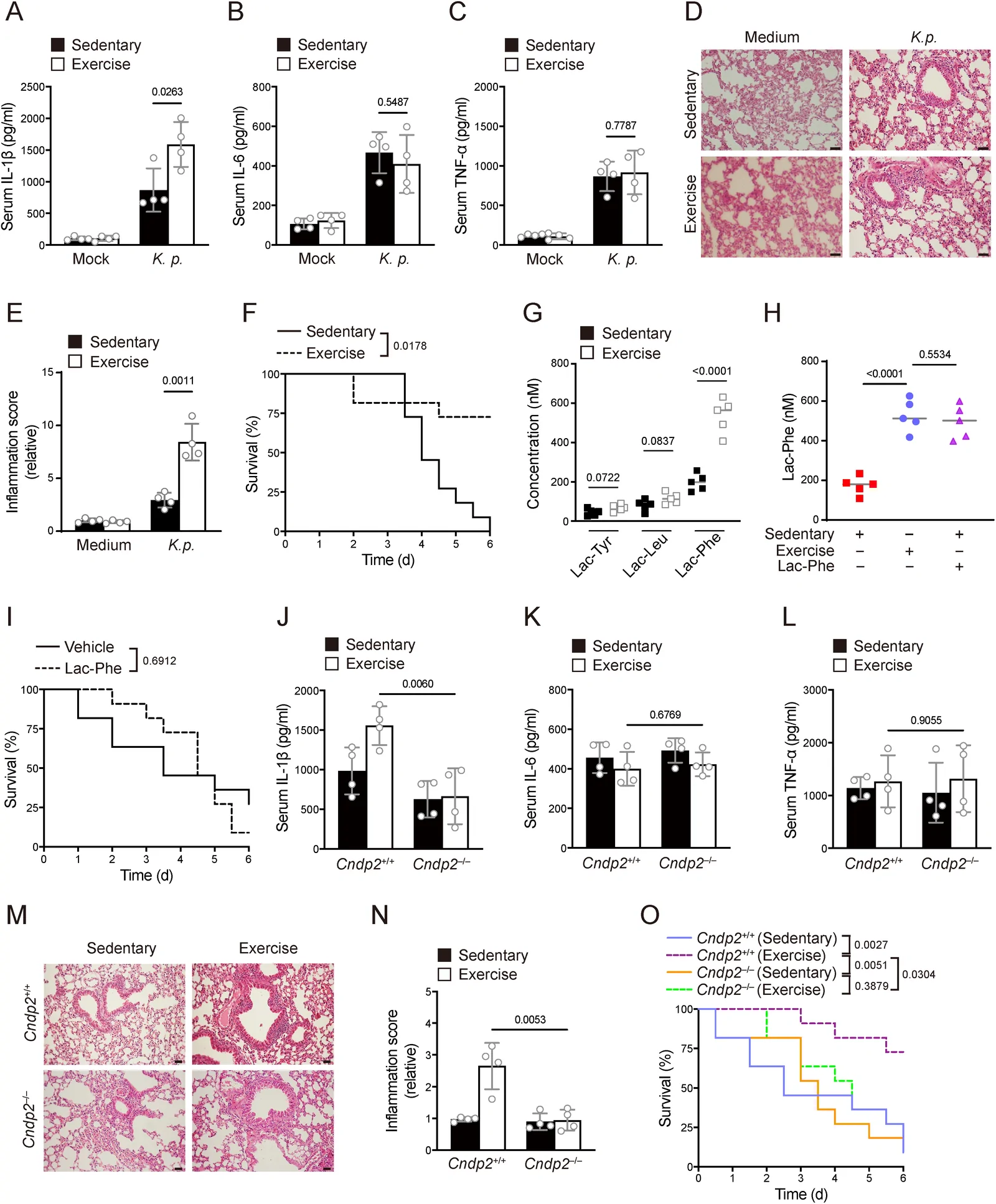
Exercise enhances the anti-infection activity. **(A–F)** Wild-type mice were housed under a sedentary state or exercised for one month, followed by intratracheal inoculation of 3 x 10^4^ CFU of *K. pneumoniae* (*K.* ***p.***). Serum levels of IL-1β **(A)**, IL-6 **(B)** and TNF-α **(C)** were determined through ELISA assays 24 h post infection. Mouse lungs were fixed and subjected to Hematoxylin and Eosin staining 24 h post infection **(D)**. Inflammation scores were calculated through comparing degree of lymphocyte infiltration around bronchus between distinct conditions **(E)**. Survivals of mice were monitored **(F)**. **(G)** Wild-type mice were housed under a sedentary state or exercised for one month. Plasma samples were collected 1 h after the final exercise session, and levels of Lac-AAs were determined through quantitative mass spectrometry. **(H)** Wild-type mice were housed under a sedentary state or exercised for one month, or intraperitoneally injected with 5 μl/g Lac-Phe per day for seven consecutive days. Plasma levels of Lac-Phe were determined through quantitative mass spectrometry. **(I)** Wild-type mice were intraperitoneally injected with 5 μl/g Lac-Phe per day for seven consecutive days. Mice were then intratracheally inoculated with 3 x 10^4^ CFU of *K. pneumoniae* (*K.* ***p.***). Survivals of mice were monitored. **(J–O)** *Cndp2*^+/+^ and *Cndp2*^–/–^ mice were housed under a sedentary state or exercised for one month, followed by intratracheal inoculation of 3 x 10^4^ CFU of *K. pneumoniae*. Serum levels of IL-1β **(J)**, IL-6 **(K)** and TNF-α **(L)** were determined through ELISA assays 24 h post infection. Mouse lungs were fixed and subjected to Hematoxylin and Eosin staining 24 h post infection **(M)**. Inflammation scores were calculated through comparing degree of lymphocyte infiltration around bronchus between distinct conditions **(N)**. Survivals of mice were monitored **(O)**. For F, I, O, n = 11. Scale bar: 100 μm. Data were shown as means±SD. Experiments were repeated three times with similar results.

Previous reports have shown that after exercise, multiple Lac-AAs are produced and enter the plasma, among which Lac-Phe is the most abundant and implicated in exercise induced physiological adaptations [19,22]. Consistently, we found that after a month of moderate exercise, Lac-Phe levels are increased and significantly higher than other Lac-AAs (Fig 1G). It is not yet clear whether Lac-Phe is related to anti-infection activity. We found that the administration of Lac-Phe which imitates exercise effect did not alter the release of pro-inflammatory cytokines in mice after infection (Fig 1H and S1A–S1C Fig). The survival rate of mice after lethal dose infection was not altered by the treatment of Lac-Phe (Fig 1I). CNDP2 is a key enzyme that produces Lac-Phe during exercise [18,19]. We found that compared with wild-type mice, *Cndp2* knockout mice that underwent exercise had significantly reduced secretion of IL-1β post infection (Fig 1J), while IL-6 or TNF-α was not affected (Fig 1K and 1L). Less lymphocyte infiltration and bacterial clearance were observed in lung tissues of *Cndp2* knockout mouse (Fig 1M, 1N and S1D Fig). Moreover, survival rate of *Cndp2* knockout mice significantly decreased upon lethal dose infection (Fig 1O). CNDP2 is highly expressed in macrophages [19]. We prepared mice with specific knockout of *Cndp2* in macrophages (S1E Fig). Compared with wild-type mice that underwent exercise, mice with macrophage *Cndp2* deficiency showed a significant reduction in the release of IL-1β after infection (S1F Fig), while IL-6 or TNF-α was not affected (S1G and S1H Fig). Lung tissue sections of *Cndp2* conditional deficient mouse showed less lymphocyte infiltration compared with wild-type controls (S1I and S1J Fig). The survival rate of *Cndp2* conditional knockout mice significantly decreased post lethal dose infection (S1K Fig). These results indicate that CNDP2 is involved in the process of enhancing anti-infection ability through exercise, and this process does not depend on Lac-Phe.

### CNDP2 controls hyperactivation-like state post exercise

To investigate how *Cndp2* deficiency affects macrophage function, we isolated peritoneal macrophages from wild-type and *Cndp2* knockout mice and co-cultured them with *K. pneumoniae*. We found that although the ability of peritoneal macrophages to secrete IL-1β decreased after *Cndp2* knockout (Fig 2A), the ability to secrete IL-6 and TNF-α was not affected (Fig 2B and 2C). Wild-type peritoneal macrophages had increased cell viability and decreased cell cytotoxicity after exercise (Fig 2D and 2E). However, this trend of change was diminished in *Cndp2* knockout peritoneal macrophages that had undergone exercise (Fig 2D and 2E).

**Fig 2:**
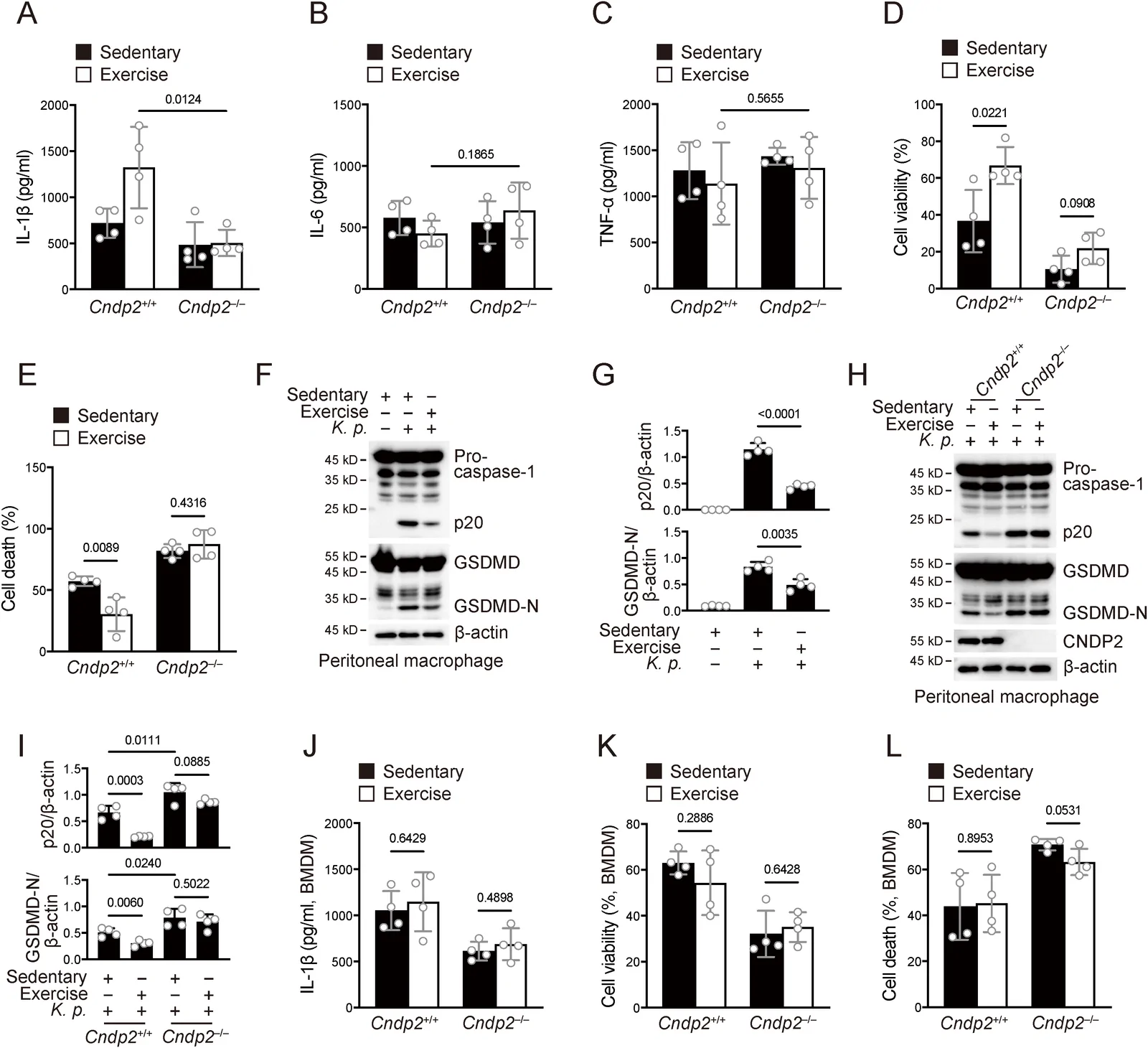
CNDP2 is involved in the establishment of inflammasome hyperactivation. **(A–E)** *Cndp2*^+/+^ and *Cndp2*^–/–^ mice were housed under a sedentary state or exercised for one month. Peritoneal macrophages were collected from *Cndp2*^+/+^ and *Cndp2*^–/–^ mice and cultured in the presence of *K. pneumoniae* at a multiplicity of infection (MOI) of 10 for 1 h. Cells were then supplemented with fresh medium containing 100 μg/ml gentamycin. Cell culture supernatants were collected for ELISA assays of IL-1β **(A)**, IL-6 **(B)** and TNF-α **(C)** 24 h later. Percentages of viable cells were determined by examining cellular ATP levels in cell pellets 24 h post infection **(D)**. Percentages of cells undergoing pyroptosis were calculated by assessing released LDH in cell culture supernatants 24 h post infection **(E)**. **(F, G)** Wild-type mice were housed under a sedentary state or exercised for one month. Peritoneal macrophages were collected from these mice and cultured in the presence of *K. pneumoniae* at an MOI of 10 for 1 h. Cells were then supplemented with fresh medium containing 100 μg/ml gentamycin. Cells were collected and immunoblotted with antibodies against the indicated proteins 6 h post infection **(F)**. Band intensities of cleaved caspase-1 (p20) and GSDMD (GSDMD-N) were calculated and shown as relative to that of β-actin **(G)**. **(H,** **I)** *Cndp2*^+/+^ and *Cndp2*^–/–^ mice were housed under a sedentary state or exercised for one month. Peritoneal macrophages were collected from these mice and cultured in the presence of *K. pneumoniae* at an MOI of 10 for 1 h. Cells were then supplemented with fresh medium containing 100 μg/ml gentamycin. Cells were collected and immunoblotted with antibodies against the indicated proteins 6 h post infection **(H)**. Band intensities of cleaved caspase-1 (p20) and GSDMD (GSDMD-N) were calculated and shown as relative to that of β-actin **(I)**. **(J–L)** *Cndp2*^+/+^ and *Cndp2*^–/–^ mice were housed under a sedentary state or exercised for one month. BMDM cells were collected from these mice and cultured in the presence of *K. pneumoniae* at an MOI of 10 for 1 h. Cells were then supplemented with fresh medium containing 100 μg/ml gentamycin. Cell culture supernatants were collected for ELISA assays of IL-1β 24 h post infection **(J)**. Percentages of viable cells were determined by examining cellular ATP levels in cell pellets 24 h post infection **(K)**. Percentages of cells undergoing pyroptosis were calculated by assessing released LDH in cell culture supernatants 24 h post infection **(L)**. Data were shown as means±SD. Experiments were repeated three times with similar results.

During infections, inflammasome mediated cell pyroptosis is a key biological process that controls the strength of inflammatory responses [2,3]. After exercise, the ability of macrophages to secrete IL-1β and cell viability increase, which is similar to the hyperactivation state of macrophages [14,28]. Indeed, inflammasome hyperactivation in peritoneal macrophages from wild-type mice that had undergone exercise was established in respect of caspase-1 activation reduction and GSDMD cleavage suppression (Fig 2F and 2G). We found that macrophages could maintain cell membrane integrity with lower PI uptake and promote IL-1β release consistently in a hyperactivation-like state (S2A and S2B Fig). After *Cndp2* knockout, the activation of inflammasomes after infection was enhanced compared to wild-type ones, but it was no longer affected by exercise (Fig 2H and 2I). The restriction of excessive inflammasome activation in mouse macrophages after exercise may be a key factor in causing macrophage hyperactivation.

We prepared wild-type and *Cndp2* knockout bone marrow-derived macrophages (BMDM) cells and examined the cellular status of inflammasomes post infection. Compared with BMDM cells from sedentary mice, the activation of inflammasomes in cells from wild-type mice that had undergone exercise was almost the same (S2C–S2G Fig). In *Cndp2* deficient cells, the activation of inflammasomes after infection was enhanced compared to wild-type ones, but it was not affected by exercise (Fig 2I and S2H Fig). Correspondingly, the ability of cells to secrete cytokines, cell viability or cell cytotoxicity were not affected by exercise (Fig 2J–2L). The data from isolated peritoneal macrophages indicate that the effect of exercise on macrophages is transient, while cultured BMDMs seem to lose this signaling, suggesting that the effect is achieved through the continuous transduction of a yet unknown signal by the body to macrophages, rather than completely altering the ability to resist infections at an epigenetic level. However, in *Nlrp3* deficient cells, exercise-induced inflammasome hyperactivation was diminished, indicating the dependence of NLRP3 in this process (S2J–S2N Fig). Together, these results indicate that CNDP2 regulates the intensity of inflammasome activation in mice after exercise.

### CNDP2 interacts with GSDMD

In order to identify the molecular mechanism by which CNDP2 regulates inflammasome hyperactivation, we used CNDP2 as a bait to screen a mouse bone marrow cDNA library. Among the screened clones, many of them encoded the GSDMD protein (Fig 3A). We validated the binding of CNDP2 to GSDMD in yeast cells (Fig 3B). We also demonstrated the binding of CNDP2 and GSDMD in HEK293T cells (S3A Fig). In wild-type BMDM cells, CNDP2 also interacted with GSDMD (Fig 3C and S3B Fig). Moreover, CNDP2 bound GSDMD in peritoneal macrophages from sedentary mice and their binding ability was enhanced in cells from exercised mice (Fig 3D–3G). However, bacterial infection does not enhance the binding of CNDP2 to GSDMD (S3C and S3D Fig). We found the N-terminus of GSDMD interacted with CNDP2 (S3E–S3G Fig) and GSDMD bound the N-terminus of CNDP2 (S3F and S3H Fig). We restored a truncated CNDP2 in *Cndp2* knockout BMDM cells and found that the truncated CNDP2 did not constrict the activation of inflammasomes in respect of cytokine release, cell viability or cell cytotoxicity (Fig 3H–3L). These results indicate that CNDP2 controls inflammasome activity during exercise through binding GSDMD.

**Fig 3:**
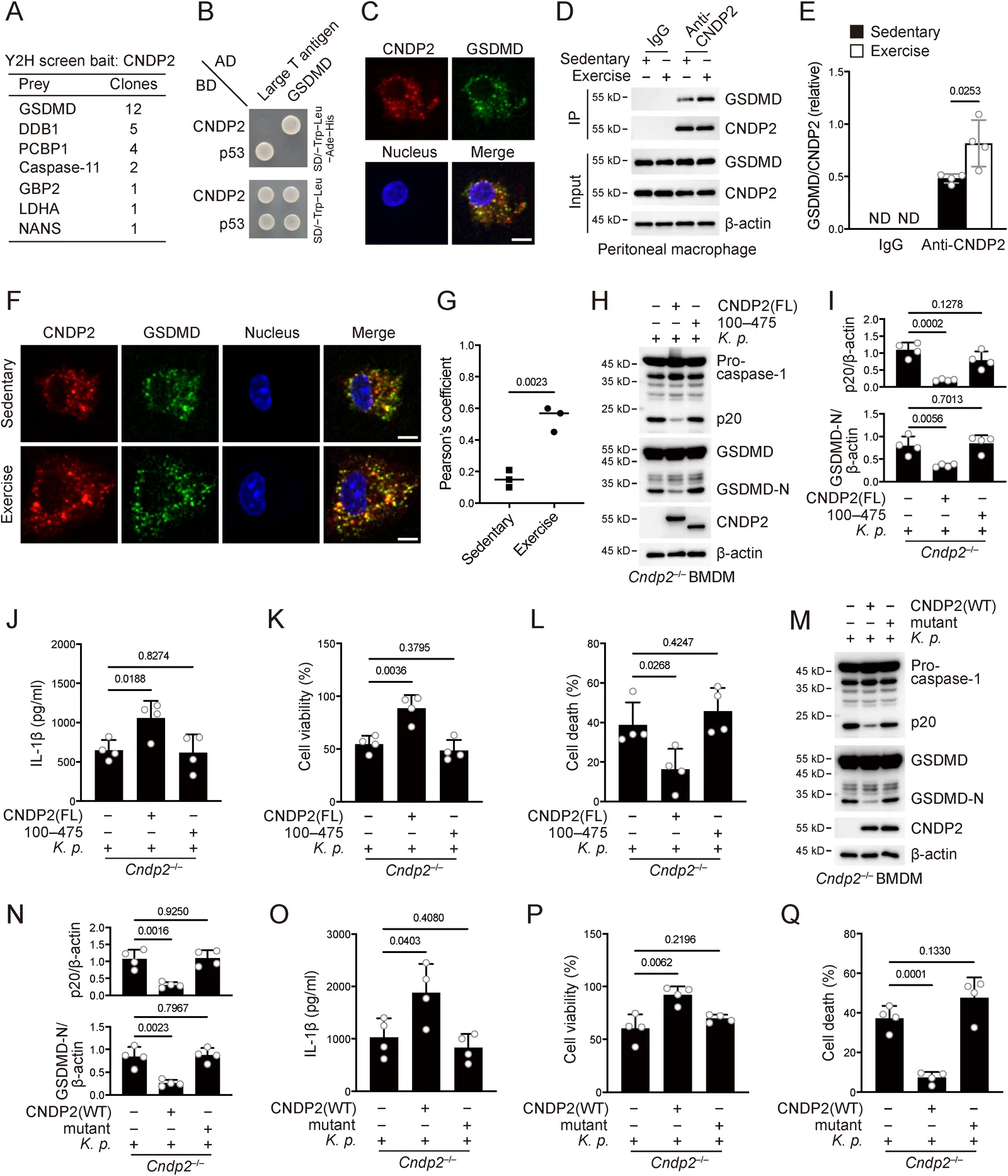
CNDP2 associates with GSDMD. **(A)** Yeast two-hybrid screening was performed using CNDP2 as a bait, and a mouse bone marrow cDNA library was screened. Positive clones and clone number were shown. **(B)** Plasmids encoding Gal4 DNA binding domain (BD)-tagged CNDP2 and Gal4 activating domain (AD)-tagged GSDMD were co-transfected into the yeast strain AH109. Transformants were grown in the indicated selection media. BD-tagged p53 and AD-tagged large T antigen were co-transfected serving as a positive control. **(C)** Wild-type BMDM cells were fixed and stained with antibodies against CNDP2 and GSDMD. Nucleus was stained with DAPI. Scale bar: 10 μm. **(D–G)** Wild-type mice were housed under a sedentary state or exercised for one month. Peritoneal macrophages were collected from these mice and subjected to immunoprecipitation with antibody against CNDP2. Precipitates were immunoblotted with antibodies against the indicated proteins **(D)**. Band intensities of precipitated GSDMD were relative to that of precipitated CNDP2 **(E)**. Peritoneal macrophages were also cultured to adhere to the wall, followed by fixing and staining with antibodies against CNDP2 and GSDMD. Nucleus was stained with DAPI. Scale bar: 10 μm **(F)**. Pearson’s coefficient of GSDMD to CNDP2 was calculated **(G)**. **(H–L)** *Cndp2*^–/–^ BMDM cells were infected with lentiviruses encoding full-length (FL) CNDP2 or the truncate for 48 h. Cells were cultured in the presence of *K. pneumoniae* at an MOI of 10 for 1 h. Cells were then supplemented with fresh medium containing 100 μg/ml gentamycin. Cells were collected and immunoblotted with antibodies against the indicated proteins 6 h post infection **(H)**. Band intensities of cleaved caspase-1 (p20) and GSDMD (GSDMD-N) were calculated and shown as relative to that of β-actin **(I)**. Cell culture supernatants were collected for ELISA assays of IL-1β 24 h post infection **(J)**. Percentages of viable cells were determined by examining cellular ATP levels in cell pellets 24 h post infection **(K)**. Percentages of cells undergoing pyroptosis were calculated by assessing released LDH in cell culture supernatants 24 h post infection **(L)**. **(M–Q)** *Cndp2*^–/–^ BMDM cells were infected with lentiviruses encoding wild-type (WT) CNDP2 or the mutant (D132A) for 48 h. Cells were cultured in the presence of *K. pneumoniae* at an MOI of 10 for 1 h. Cells were then supplemented with fresh medium containing 100 μg/ml gentamycin. Cells were collected and immunoblotted with antibodies against the indicated proteins 6 h post infection **(M)**. Band intensities of cleaved caspase-1 (p20) and GSDMD (GSDMD-N) were calculated and shown as relative to that of β-actin **(N)**. Cell culture supernatants were collected for ELISA assays of IL-1β 24 h post infection **(O)**. Percentages of viable cells were determined by examining cellular ATP levels in cell pellets 24 h post infection **(P)**. Percentages of cells undergoing pyroptosis were calculated by assessing released LDH in cell culture supernatants 24 h post infection **(Q)**. Data were shown as means±SD. Experiments were repeated three times with similar results.

We speculate that CNDP2 might inhibit the cleavage of GSDMD after binding, thereby indirectly affecting the cleavage of caspase-1 [29]. However, adding CNDP2 to an *in vitro* cleavage system did not affect the cleavage of GSDMD by caspase-1 (S3I and S3J Fig). Moreover, there was no interaction between CNDP2 and caspase-1 (S3K Fig). Whether the restriction of inflammasome activation by CNDP2 was related to its enzymatic activity was the next question we were concerned about. We restored *Cndp2* deficient cells with CNDP2 mutants. The activation of inflammasomes cannot be suppressed in cells rescued with CNDP2 mutations (Fig 3M and 3N). The release of cytokines, cell viability or cell cytotoxicity were also not affected by CNDP2 mutants (Fig 3O–3Q). Since Lac-Phe does not affect inflammasome activation, we speculate whether CNDP2 regulated inflammasomes by hydrolyzing the dipeptide Cys-Gly. We transfected Cys-Gly into *Cndp2* knockout cells and found that Cys-Gly did not affect the activation of inflammasomes post infection (S3L and S3M Fig). Moreover, the release of cytokines, cell viability or cell cytotoxicity were not affected by Cys-Gly transfection (S3N–S3P Fig). These data suggest that the establishment of inflammasome hyperactivation-like state by CNDP2 may be achieved through an unknown and enzymatic activity-dependent process.

### CNDP2 catalyzes the production of γ-Glu-Cys

We accidentally found that the ability of Glutathione Sepharose resin incubated with His-CNDP2 and GSDMD N-terminus had a decreased binding ability to GST proteins, and this phenomenon only occurred when both CNDP2 and GSDMD N-terminus were present (S4A and S4B Fig). The enzymatic activity-mutated CNDP2 cannot affect the binding of Glutathione Sepharose resin to GST proteins anymore (S4C and S4D Fig). Due to the ability of CNDP2 to hydrolyze dipeptides Cys-Gly and GSH on Glutathione Sepharose resins contain Cys-Gly, we surmised that CNDP2 hydrolyzed GSH. We incubated CNDP2, GSDMD N-terminus and GSH together and identified the catalytic products by mass spectrometry (Fig 4A). We found that when CNDP2 and GSDMD N-terminus co-exist, GSH was hydrolyzed into γ-Glu-Cys (Fig 4A and 4B).

**Fig 4:**
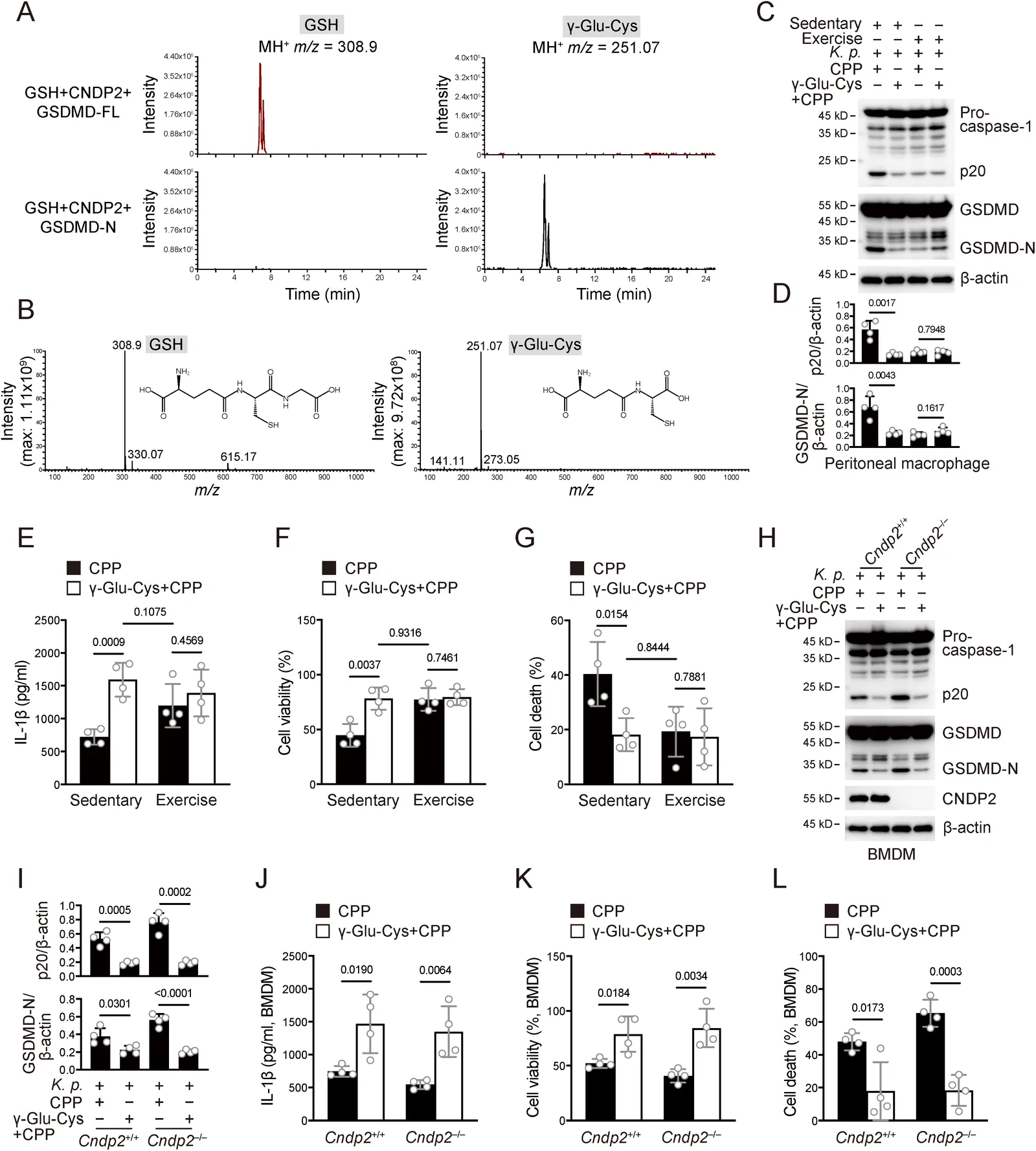
CNDP2 catalyzes γ-Glu-Cys production. **(A, B)** GSH was incubated with recombinant CNDP2, GSDMD full-length or GSDMD N-terminus, followed by HPLC fractionation **(A)**. Main components were identified by mass spectrometry **(B)**. **(C–G)** Wild-type mice were housed under a sedentary state or exercised for one month. Peritoneal macrophages were collected from these mice and transfected with 10 mM γ-Glu-Cys with the help of cell-penetrating peptides for 6 h. Cells were further cultured in the presence of *K. pneumoniae* at an MOI of 10 for 1 h. Cells were then supplemented with fresh medium containing 100 μg/ml gentamycin. Cells were collected and immunoblotted with antibodies against the indicated proteins 6 h post infection **(C)**. Band intensities of cleaved caspase-1 (p20) and GSDMD (GSDMD-N) were calculated and shown as relative to that of β-actin **(D)**. Cell culture supernatants were collected for ELISA assays of IL-1β 24 h post infection **(E)**. Percentages of viable cells were determined by examining cellular ATP levels in cell pellets 24 h post infection **(F)**. Percentages of cells undergoing pyroptosis were calculated by assessing released LDH in cell culture supernatants 24 h post infection **(G)**. **(H–L)** *Cndp2*^+/+^ and *Cndp2*^–/–^ BMDM cells were collected from these mice and transfected with 10 mM γ-Glu-Cys with the help of cell-penetrating peptides for 6 h. Cells were further cultured in the presence of *K. pneumoniae* at an MOI of 10 for 1 h. Cells were then supplemented with fresh medium containing 100 μg/ml gentamycin. Cells were collected and immunoblotted with antibodies against the indicated proteins 6 h post infection **(H)**. Band intensities of cleaved caspase-1 (p20) and GSDMD (GSDMD-N) were calculated and shown as relative to that of β-actin **(I)**. Cell culture supernatants were collected for ELISA assays of IL-1β 24 h post infection **(J)**. Percentages of viable cells were determined by examining cellular ATP levels in cell pellets 24 h post infection **(K)**. Percentages of cells undergoing pyroptosis were calculated by assessing released LDH in cell culture supernatants 24 h post infection **(L)**. Data were shown as means±SD. Experiments were repeated three times with similar results.

To find whether the establishment of inflammasome hyperactivation-like state during exercise was related to γ-Glu-Cys, we collected peritoneal macrophages and transfected γ-Glu-Cys into these cells to examine its effects on infection. γ-Glu-Cys transfection induced inflammasome hyperactivation in terms of restricted excessive inflammasome activation (Fig 4C and 4D), increased inflammatory cytokine release (Fig 4E), increased cell viability and decreased cell cytotoxicity in cells from sedentary mice (Fig 4F and 4G), which was comparable to that of cells from exercised mice (Fig 4C–4G). In cells from exercised mice, the effect of γ-Glu-Cys transfection was limited (Fig 4C–4G), suggesting that CNDP2-mediated γ-Glu-Cys production may be a key factor in exercise-induced macrophage hyperactivation. In addition, in *Cndp2* knockout peritoneal macrophages, transfection of γ-Glu-Cys suppressed excessive inflammasome activation and induced the establishment of inflammasome hyperactivation with limited pyroptotic cell death (S4E–S4I Fig). Indeed, exercise increased the amount of GSH (S4J and S4K Fig), but compared to the decrease in intracellular GSH at the millimolar level, the amount of γ-Glu-Cys generated by CNDP2 was at the micromolar level (S4K and S4L Fig). This indicates that the decrease in GSH during the infection process was not caused by CNDP2. Consistently, we used the inhibitor of CNDP2 (bestatin), however it did not decrease intracellular γ-Glu-Cys level (S4M Fig), suggesting that the active center of CNDP2 catalyzing γ-Glu-Cys may differ from the classical enzyme active site, or the binding of CNDP2 to GSDMD N-terminus may hinder its contact with inhibitors.

In *Cndp2* knockout BMDM cells, γ-Glu-Cys transfection also induced inflammasome hyperactivation (Fig 4H–4L). Accordingly, the intracellular levels of γ-Glu-Cys were elevated in wild-type cells incubated with γ-Glu-Cys post infection (S4N Fig). Indeed, the intracellular GSH levels both increased in wild-type and *Cndp2* deficient cells incubated with γ-Glu-Cys (S4O Fig), further suggesting that CNDP2 itself does not affect the uptake of γ-Glu-Cys. However, if γ-Glu-Cys was added to the culture medium, although it constricted excessive inflammasome activation in wild-type cells, it could not restrict inflammasome activation in *Cndp2* deficient cells (S5A–S5E Fig). This phenomenon might be explained by that the addition of extracellular γ-Glu-Cys increased the intracellular GSH content through the GSH synthesis pathway [26,27], thereby elevating the intracellular γ-Glu-Cys level through CNDP2 hydrolyzing. In summary, these results indicate that CNDP2-mediated γ-Glu-Cys production is a key factor in the establishment of inflammasome hyperactivation-like state during infections.

### γ-Glu-Cys protects mitochondrial membrane integrity through inhibiting CypD

To investigate how γ-Glu-Cys controls inflammasome hyperactivation with limited pyroptotic cell death, we took use of a screening system in which cells infected with sgRNA-containing lentiviruses were incubated with *K. pneumoniae*, followed by examination of secreted cytokines (Fig 5A and 5B). In top-ranked candidates, we further examined whether proteins encoded by these candidate genes bound γ-Glu-Cys (S5F Fig). Post screening, CypD aroused our interest (Fig 5C). CypD is a mitochondrial protein that participates in the formation of mitochondrial permeability transition pore under physiological conditions [30]. The ability of *Ppif* (the gene encoding CypD protein) knockout mice to secrete IL-1β was enhanced after infection (S5G Fig), with an increase in infiltrating lymphocytes and a decrease in bacterial load in mouse lung tissues, which induced a significant improvement in mice survival (Fig 5D–5G). However, the production of γ-Glu-Cys was not affected by *Ppif* deficiency (S5H Fig). The enhancement of the anti-infection ability was not affected by exercise factors anymore in *Ppif* knockout mice (Fig 5D–5G and S5G Fig).

**Fig 5:**
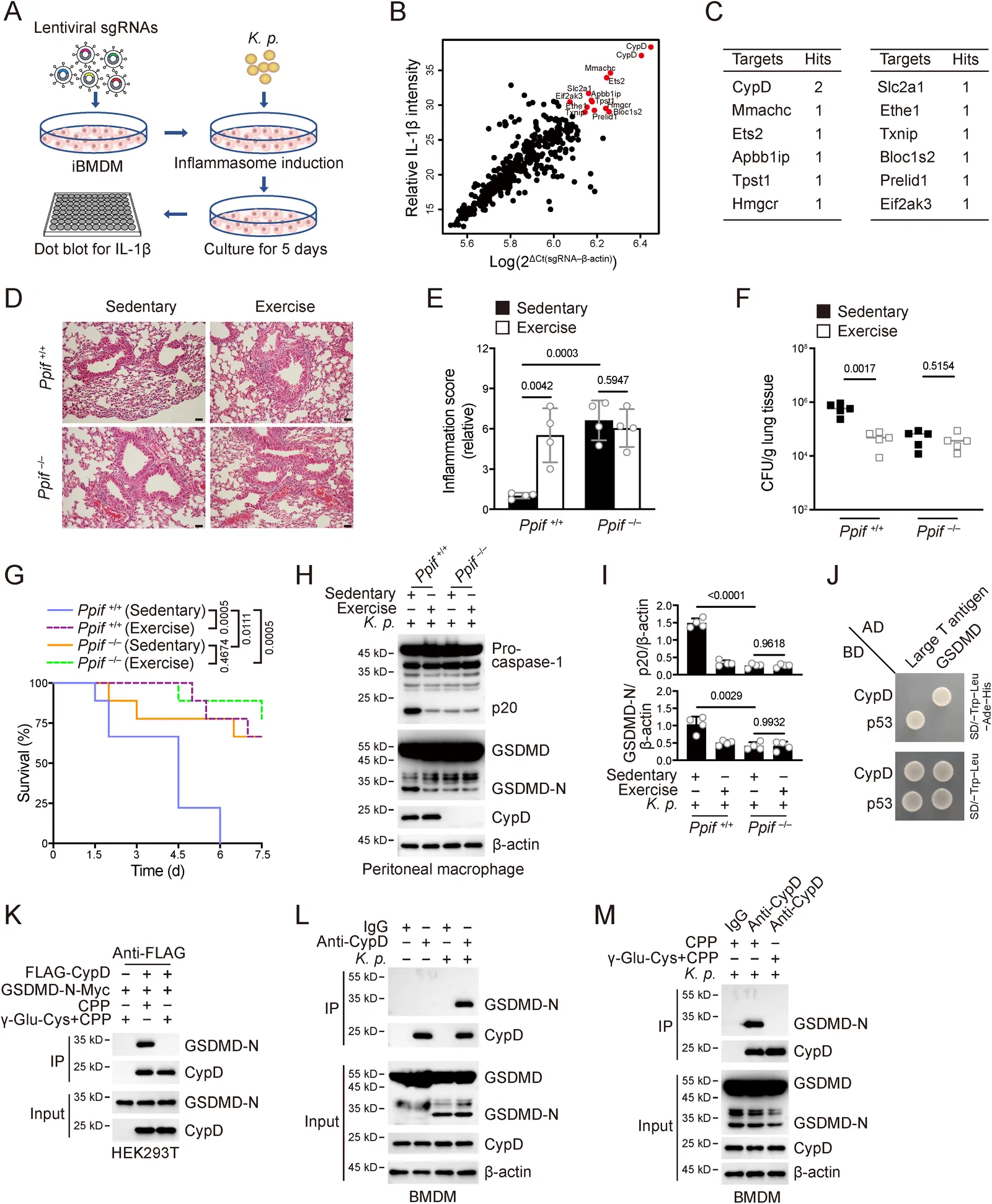
γ-Glu-Cys inhibits CypD binding to GSDMD N-terminus. **(A–C)** A scheme of CRISPR/Cas9-based screening for factors involved in inflammasome hyperactivation was shown. Briefly, iBMDM cells were infected lentiviruses carrying Cas9 and sgRNAs, followed by infection of *K. pneumoniae*. Cells were cultured for 5 days and supernatants were transferred to membranes through a dot-blot transferring system **(A).** Cleaved IL-1β signals were quantified and top-ranked cells were also quantified for their sgRNA abundance **(B).** Targets of interest were listed **(C).** **(D–G)** *Ppif*^+/+^ and *Ppif*^–/–^ mice were housed under a sedentary state or exercised for one month, followed by intratracheal inoculation of 3 x 10^4^ CFU of *K. pneumoniae*. Mouse lungs were fixed and subjected to Hematoxylin and Eosin staining 24 h post infection **(D).** Inflammation scores were calculated through comparing degree of lymphocyte infiltration around bronchus between distinct conditions **(E).** Bacterial load was examined in mouse lung tissues post infection **(F).** Survivals of mice were monitored **(G).** **(H, I)** *Ppif*^+/+^ and *Ppif*^–/–^ mice were housed under a sedentary state or exercised for one month. Peritoneal macrophages were collected from these mice and cultured in the presence of *K. pneumoniae* at an MOI of 10 for 1 h. Cells were then supplemented with fresh medium containing 100 μg/ml gentamycin. Cells were collected and immunoblotted with antibodies against the indicated proteins 6 h post infection **(H).** Band intensities of cleaved caspase-1 (p20) and GSDMD (GSDMD-N) were calculated and shown as relative to that of β-actin **(I).** **(J)** Plasmids encoding Gal4 DNA binding domain (BD)-tagged CypD and Gal4 activating domain (AD)-tagged GSDMD were co-transfected into the yeast strain AH109. Transformants were grown in the indicated selection media. BD-tagged p53 and AD-tagged large T antigen were co-transfected serving as a positive control. **(K)** Plasmids encoding CypD and GSDMD N-terminus were co-transfected into HEK293T cells for 10 h. Cells were then transfected with 10 mM γ-Glu-Cys with the help of cell-penetrating peptides for 6 h, followed by immunoprecipitation with an antibody against FLAG. Precipitates were immunoblotted with antibodies against the indicated proteins. **(L)** BMDM cells were collected from wild-type mice and cultured in the presence of *K. pneumoniae* at an MOI of 10 for 1 h. Cells were then supplemented with fresh medium containing 100 μg/ml gentamycin. Cells were collected and immunoprecipitated with a control IgG or antibody against CypD 6 h post infection, followed by immunoblotting with antibodies against the indicated proteins. **(M)** Wild-type BMDM cells were transfected with 10 mM γ-Glu-Cys with the help of cell-penetrating peptides for 6 h. Cells were further cultured in the presence of *K. pneumoniae* at an MOI of 10 for 1 h. Cells were then supplemented with fresh medium containing 100 μg/ml gentamycin. Cells were collected and immunoprecipitated with a control IgG or antibody against CypD 1 h post infection, followed by immunoblotting with antibodies against the indicated proteins. For G, n = 9. Scale bar: 100 μm. Data were shown as means±SD. Experiments were repeated three times with similar results.

We also isolated wild-type and *Ppif* knockout peritoneal macrophages and incubated them with *K. pneumoniae*. After *Ppif* knockout, the activation of inflammasomes in peritoneal macrophages was suppressed to a lower level (Fig 5H and 5I), but the ability to secrete IL-1β was increased accompanied with increased cell viability and decreased cell cytotoxicity (S5I–S5K Fig). This trend of change was not affected by exercise (Fig 5H, 5I and S5I–S5K Fig). We screened for the binding protein of CypD through yeast two-hybrid and found that CypD interacted with GSDMD (Fig 5J and S6A Fig). We validated the binding of CypD to GSDMD N-terminus in HEK293T cells (S6B and S6C Fig). The C-terminus of CypD was responsible for GSDMD binding (S6B and S6D Fig). γ-Glu-Cys inhibited the binding of CypD to GSDMD N-terminus (Fig 5K). CypD bound GSDMD N-terminus during bacterial infection (Fig 5L), while γ-Glu-Cys inhibited this binding (Fig 5M). We docked γ-Glu-Cys into CypD structure and found key sites for their binding (S6E Fig). Mutating one of the key residues significantly inhibited the binding of CypD and γ-Glu-Cys (S6F Fig).

Previous reports have suggested that GSDMD N-terminus can form pores in the inner and outer membranes of mitochondria, causing leakage of mitochondrial DNA and further exacerbating the activation of inflammasomes [12,13]. We found that when CypD was absent, mitochondrial DNA release was inhibited (S6G Fig), suggesting that pores formation on mitochondrial inner membrane was prohibited. The restoration of CypD truncations that could not be bound by GSDMD N-terminus did not induce mitochondrial DNA release either (S6H Fig). Extracellular addition of γ-Glu-Cys increases intracellular GSH levels and thus increase cytosolic γ-Glu-Cys levels through CNDP2. Indeed, incubation of γ-Glu-Cys inhibited the release of mitochondrial DNA in wild-type BMDM cells (S6I Fig). However, in *Ppif* knockout cells, the effect of γ-Glu-Cys incubation on mitochondrial DNA release was eliminated (S6I Fig). These results indicate that γ-Glu-Cys inhibits the binding of CypD and GSDMD on the inner membrane of mitochondria, thereby suppressing mitochondrial DNA release and controlling the degree of inflammasome activation.

### γ-Glu-Cys enhances the anti-infection activity post exercise

Mice were orally administered with γ-Glu-Cys to examine the *in vivo* effects of γ-Glu-Cys on inflammasome hyperactivation. We found that feeding γ-Glu-Cys specifically increased the release of IL-1β in mice, lymphocyte infiltration and bacterial clearance in mouse lung tissues (S7A–S7D Fig), and significantly improved the survival of mice after lethal dose infection (S7E Fig). Moreover, *Ppif* knockout mice had more IL-1β secretion than wild-type controls (S7A Fig). There was more lymphocyte infiltration and less bacterial load in lung tissues of *Ppif* knockout mice (S7B–S7D Fig), and the survival was also increased post infection (S7E Fig). Incubation of γ-Glu-Cys with wild-type macrophages promoted the establishment of inflammasome hyperactivation-like state (Fig 6A, 6B and S7F–S7H Fig), but after *Ppif* knockout, macrophages were already in a hyperactive state, which constrained the excessive activation of inflammasomes, increased cytokine secretion, and reduced cell mortality (Fig 6A, 6B and S7F–S7H Fig). On the contrary, in *Cndp2* knockout mice, the anti-infection ability was not affected by the administration of γ-Glu-Cys anymore (Fig 6C–6F and S7I Fig). Accordingly, intracellular γ-Glu-Cys levels were only increased in wild-type cells (S7J Fig), further confirming our surmise that extracellular γ-Glu-Cys increases cytosolic GSH levels which are hydrolyzed by CNDP2 to generate intracellular γ-Glu-Cys during this process.

**Fig 6:**
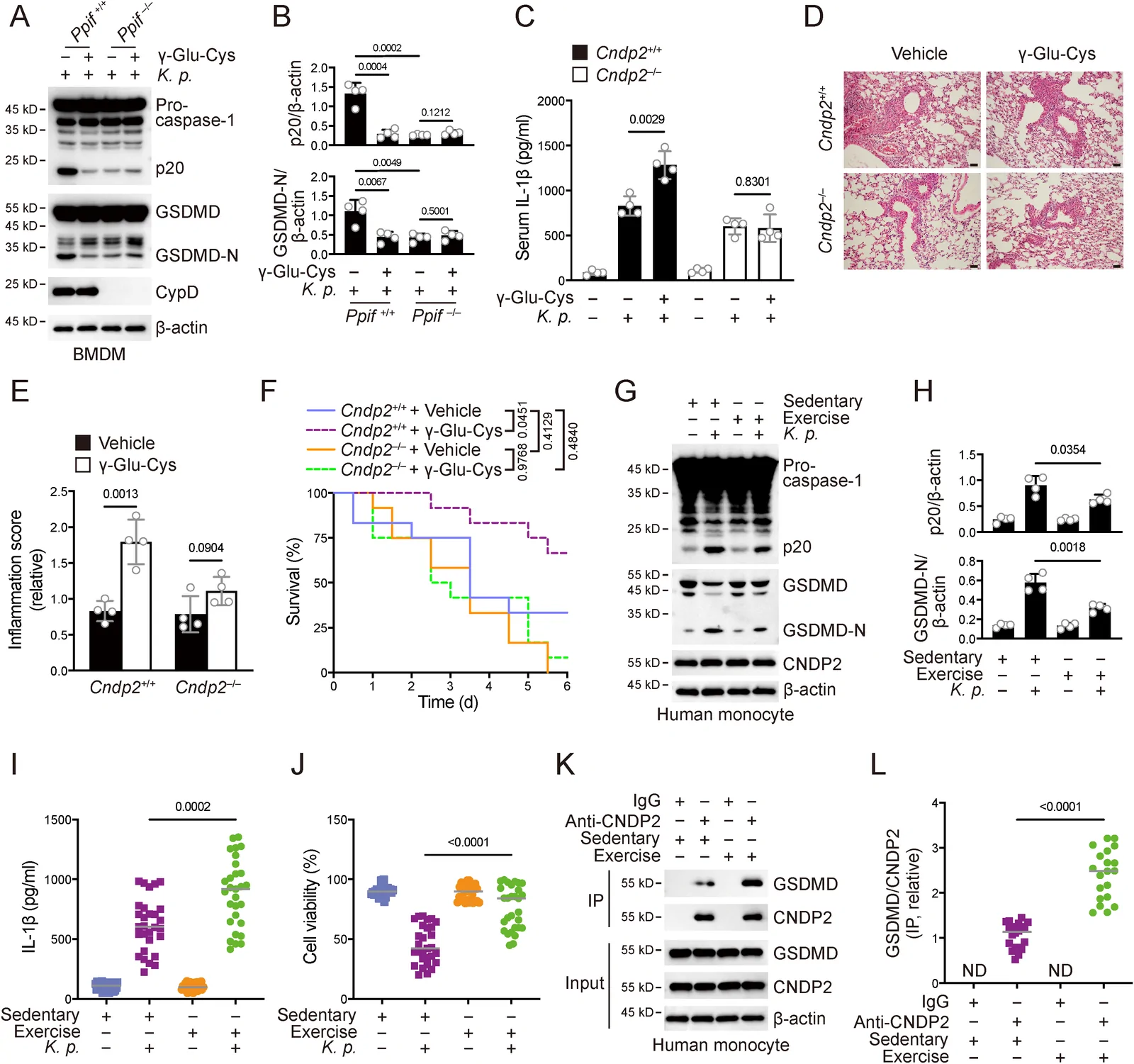
γ-Glu-Cys favors the establishment of inflammasome hyperactivation. **(A, B)** *Ppif*^+/+^ and *Ppif*^–/–^ BMDM cells were incubated with 10 mM γ-Glu-Cys for 6 h. Cells were further cultured in the presence of *K. pneumoniae* at an MOI of 10 for 1 h. Cells were then supplemented with fresh medium containing 100 μg/ml gentamycin. Cells were collected and subjected to immunoblotting with antibodies against the indicated proteins 6 h post infection **(A).** Band intensities of cleaved caspase-1 (p20) and GSDMD (GSDMD-N) were calculated and shown as relative to that of β-actin **(B).** **(C–F)** *Cndp2*^+/+^ and *Cndp2*^–/–^ mice were orally administered with 500 mg/kg γ-Glu-Cys per day for five consecutive days, followed by intratracheal inoculation of 3 x 10^4^ CFU of *K. pneumoniae*. Serum levels of IL-1β were determined through ELISA assays 24 h post infection **(C).** Mouse lungs were fixed and subjected to Hematoxylin and Eosin staining 24 h post infection **(D).** Inflammation scores were calculated through comparing degree of lymphocyte infiltration around bronchus between distinct conditions **(E).** Survivals of mice were monitored **(F).** **(G–J)** Human monocytes were isolated from bloods of healthy volunteers undergoing exercise or sedentary during the past month. Cells were further cultured in the presence of *K. pneumoniae* at an MOI of 10 for 1 h. Cells were then supplemented with fresh medium containing 100 μg/ml gentamycin. Cells were collected and subjected to immunoblotting with antibodies against the indicated proteins 6 h post infection **(G).** Band intensities of cleaved caspase-1 (p20) and GSDMD (GSDMD-N) were calculated and shown as relative to that of β-actin **(H).** Cell culture supernatants were collected for ELISA assays of IL-1β 24 h post infection **(I).** Percentages of viable cells were determined by examining cellular ATP levels in cell pellets 24 h post infection **(J).** **(K, L)** Human monocytes were isolated from bloods of healthy volunteers undergoing exercise or sedentary during the past month. Cells were immunoblotted with a control IgG or antibody against CNDP2. Precipitates were immunoblotted with antibodies against the indicated proteins **(K).** Band intensities of precipitated GSDMD were relative to that of precipitated CNDP2 **(L).** For F, n = 12; for I, J, n = 29; for L, n = 20. Scale bar: 100 μm. Data were shown as means±SD. Experiments were repeated three times with similar results.

Finally, we collected monocytes from sedentary donors or ones who exercised regularly, and examined the anti-infection activity of these cells. We found that monocytes in the exercised population were more likely to establish a hyperactive state, which inhibited the activation intensity of inflammasomes, increased cytokine secretion, decreased bacterial load and reduced cell mortality (Fig 6G–6J and S7K, S7L Fig). Intracellular binding between CNDP2 and GSDMD was enhanced in exercised human monocytes compared to sedentary cells (Fig 6K and 6L).

These results indicate that exercise can promote host’s anti-infection activity through inflammasome hyperactive state mediated by γ-Glu-Cys, a metabolite of CNDP2.

## Discussion

Here, we have discovered the molecular mechanism by which exercise can enhance the body’s ability to resist infections. During the process of inflammasome activation, pore formation by the GSDMD N-terminus on mitochondria promotes the release of mitochondrial DNA and further enhances the intensity of inflammasome activation. Exercise can enhance the binding of CNDP2 and GSDMD within macrophages. After inflammasome activation, GSDMD N-terminus together with CNDP2 promotes the conversion of GSH to γ-Glu-Cys. γ-Glu-Cys binds CypD and inhibits the binding of CypD to the GSDMD N-terminus, thereby avoiding mitochondrial inner membrane permeabilization and reducing mitochondrial DNA release.

Inflammasome hyperactivation is a physiological form of inflammasome activation that controls the degree of cell death and the duration of inflammatory cytokine secretion by reducing membrane permeabilization [28,31,32]. It prolongs the activation duration of inflammasomes and plays a crucial role in anti-infection processes [33,34]. However, the mechanism of inflammasome hyperactivation is not yet fully understood. Mitochondrial DNA release is crucial for enhancing inflammasome activation [35,36]. GSDMD N-terminus penetrates the outer membrane of mitochondria by binding to cardiolipin [13,37]. However, mitochondria are organelles with bilayer membranes, and to induce mitochondrial DNA release, GSDMD N-terminus also needs to form pores on the inner membrane of mitochondria [13,38]. CypD is likely to promote the final permeability of the mitochondrial membrane by recruiting GSDMD N-terminus onto the inner membrane of mitochondria. Exercise induces the establishment of inflammasome hyperactive state during infection by promoting the generation of γ-Glu-Cys within cells. CypD is a mitochondrial protein, and its binding to γ-Glu-Cys requires pore formation on the outer membrane of mitochondria. This activation sequence provides checkpoints for controlling inflammasome activation, giving cells more choices to determine the intensity and duration of inflammasome activation. Moreover, exercise has been shown to reduce NLRP3 inflammasome activation and IL-1β release in various disease models, including obesity, atherosclerosis, Parkinson’s disease [39–41]. In such contexts, chronic inflammasome activation contributes to sustained tissue injury, making inflammasome suppression beneficial. In contrast, during infection, inflammasome derived cytokines such as IL-1β play essential roles in antimicrobial host defense. Under these conditions, complete suppression of inflammasome activity may compromise pathogen clearance. Our data suggest that exercise induced CNDP2 and γ-Glu-Cys could reshape its downstream consequences by limiting mitochondrial damage and pyroptotic cell death while preserving inflammatory cytokine production. Future studies are warranted to dissect how exercise induce differentially modulates inflammasome activity depending on the underlying pathological mechanism.

The process of exercise generates new metabolites, but the types and effects of these products are not totally elucidated [42]. We find that CNDP2 induced Lac-Phe is more abundant in the plasma after exercise than other Lac-AAs and has been characterized exercise induced Lac-AA in previous studies [18,19]. However, Lac-Phe administration did not recapitulate the anti-infection effect of exercise, and the biological functions of other Lac-AAs especially their roles in inflammasome regulation remain to be investigated. We have discovered a new metabolite of CNDP2, γ-Glu-Cys. Although previous reports suggested that γ-Glu-Cys has anti-inflammatory effects, its mechanism is not clear yet [27,43,44]. We find that γ-Glu-Cys promotes the hyperactivation of inflammasomes by inhibiting the binding of CypD to GSDMD N-terminus. However, the inhibitor of CNDP2 for blocking γ-Glu-Cys production needs to be developed. The establishment of a hyperactive status of inflammasomes induces more persistent and increased secretion of inflammatory cytokines, which also explains why appropriate exercise is beneficial for the anti-infection activity. We obtain human monocytes from volunteers and find results consistent with our experimental findings in mouse and cellular models, which is supportive translational evidence. Future studies with larger cohorts and more sensitive single cell approaches will be required to establish the impact of exercise on inflammasome output programs in human immune cells. Moreover, whether there are other new metabolites emerge during exercise and whether these metabolites have a role in combating infections will be an important topic for the future research.

In summary, we found that the γ-Glu-Cys generated by CNDP2 during exercise promotes host’s anti-infection activity.

## Materials and methods

### Ethics statement

Animal experiments complied with relevant ethical regulations and all protocols were approved by the Biomedical Ethics Committee of Peking University (LA2022080). For human participants, written consents were obtained from all donors. Study was conducted in strict accordance with the institutional guidelines and licensed by the Ethics Committee of Peking University Third Hospital (IRB00006761-M2024837).

### Antibodies and reagents

Antibodies against IL-1β (12703, 12426), cleaved IL-1β (83186, 63124), GSDMD (93709) and caspase-1 (3866, 24232, 2225) were purchased from Cell Signaling Technology. Antibodies against caspase-1 (ab207802), CNDP2 (ab241126), CypD (ab231155, ab110324), and GSDMD (ab215203) were purchased from Abcam. Antibodies against CNDP2 (PA5–87856, PA5–87101) and antibody against CypD (45–5900) were from ThermoFisher Scientific. Anti-c-Myc magnetic beads (88843) were purchased from Pierce. Antibody against 6xHis tag (MA121315) was purchased from Invitrogen. Antibodies against FLAG tag (F3165), β-actin (A1978) and anti-FLAG M2 magnetic beads (M8823) were purchased from Sigma-Aldrich. Antibodies against c-Myc (sc-40) and GST (sc-138) were purchased from Santa Cruz Biotechnology. Anti-HA tag antibody (HX1820) was purchased from Huaxingbio (Beijing). HRP-conjugated goat anti-mouse IgG (SA00001–1) and HRP-conjugated goat anti-rabbit IgG (SA00001–2) were purchased from Proteintech Group. Alexa Fluor Plus 488-conjugated goat anti-mouse IgG (A32723) and Alexa Fluor 594-conjugated goat anti-rabbit IgG (A32740) were purchased from Invitrogen. Alexa Fluor Plus 488-conjugated donkey anti-goat IgG (bs-0294D-AF488) and Alexa Fluor 594-conjugated donkey anti-rabbit IgG (bs-0295D-AF594) were purchased from Bioss (Beijing).

Glutathione sepharose 4B resin (17075601) was purchased from Cytiva. Protein A/G PLUS-Agarose beads (sc-2003) were purchased from Santa Cruz Biotechnology. DAPI (2879038) was purchased from PeproTech (BioGems). SMART MMLV reverse transcriptase (639524) was purchased from Clontech, Takara Bio. Random primer (Hexadeoxyribonucleotide mixture, pd(N)6) (3801) was purchased from Takara Bio. Protease inhibitor cocktail (11697498001) was purchased from Roche. Total RNA extraction kit (8034111) was purchased from Dakewe (Beijing). Ni-NTA agarose beads (R90115) and AminoLink coupling resin (20381) were purchased from Invitrogen. LPS (B46894) was purchased from Innochem (Beijing). Human M-CSF (300–25) and murine M-CSF (315–02) were purchased from PeproTech. CellTiter-Glo luminescent cell viability assay kit (G7570) and CytoTox 96 non-radioactive cytotoxicity assay kit (G1780) were purchased from Promega. The following kits were used: Human Monocyte Nucleofector Kit (Lonza Cat# VPA-1007), Total RNA extraction kit (Dakewe Cat# 8034111), Mitochondrial DNA isolation kit (Abcam Cat# ab65321), Cell fractionation kit (Abcam Cat# ab109719), Mitochondrion isolation kit (Abcam Cat# ab110170), Mitochondrion isolation kit (Invitrogen Cat# 89874). Cell-penetrating peptides (ab142343) were purchased from Abcam. ELISA kits for detecting mouse IL-1β (D721071), TNF-α (D721026) and IL-6 (D721022) were obtained from BBI Life Sciences (Shanghai).

### Mice and infection

*Cndp2*^flox/flox^ (S-CKO-12850), *Cndp2*^–/–^ (S-KO-11504), *Ppif*^–/–^ (S-KO-00416) and *Lyz2*-Cre (C001358) mice were purchased from Cyagen Biosciences (Jiangsu, China). *Cndp2*^flox/flox^ mice were crossed with *Lyz2*-Cre mice to generate a *Cndp2* conditional knockout strain. Peritoneal macrophages were collected from *Cndp2*^flox/flox^;*Lyz2*-Cre mice. Mice were intratracheally inoculated with 3 x 10^4^ CFU of *K. pneumoniae* (*K. p.*). Survivals of mice were monitored in the following days post infection. Serum levels of inflammatory cytokines were examined through ELISA assays 24 h post infection.

### Bacteria and CFU determination

*K. pneumoniae* serotype 2 strain (43816) was used [45]. Bacteria were preserved in LB plates and inoculated into liquid LB medium, followed by shaking at 37 °C for 16 h. Bacteria either from culture or tissue homogenates were diluted and spread into LB plates to determine the CFU numbers.

### Exercise model and γ-Glu-Cys administration

Mice were placed in a six-channel mouse treadmill, and after 5 minutes of adaptation, the treadmill began to run. The inclination angle of the treadmill was set to 5 degrees, and the starting speed was 6 meters per minute. Subsequently, every 5 minutes, the speed of the treadmill increased by 2 meters per minute until the maximum speed reached 20 meters per minute. The mouse exercised on the treadmill for one hour. The time for exercise was from 2 pm to 4 pm every day, five days a week from Monday to Friday, and no exercise on weekends. Exercise lasted for a month. On the second morning after the end of exercise, the mice were intratracheally inoculated with 3 x 10^4^ CFU of *K. pneumoniae* (*K. p.*). If it was to detect cytokines in plasma, the blood collection time was 24 hours after infection or 96 hours after infection. Similarly, on the morning after exercise, the bone marrow of mice was collected for BMDM preparation, or the peritoneal macrophages of mice were collected. Mice were orally administered with 500 mg/kg γ-Glu-Cys per day for five consecutive days, followed by intratracheal inoculation of 3 x 10^4^ CFU of *K. pneumoniae*. After infection, mice were still fed water containing γ-Glu-Cys. Serum levels of IL-1β were determined through ELISA assays 24 h post infection.

### Primary cell separation and culture

To generate BMDM cells, bone marrow cells were flushed from mouse femurs and tibias, followed by red blood cell removal using red blood cell lysis buffer. Cells were resuspended in alpha-MEM containing 10% (v/v) fetal bovine serum and 10 ng/ml M-CSF and grown at 37 °C with a 5% CO_2_ humidified atmosphere for 7 days.

To produce human macrophages, human bloods collected from healthy donors were mixed with an equal volume of saline and then added to lymphocyte separation medium, followed by centrifugation and PBMC collection. Human PBMCs were incubated with APC-conjugated anti-CD14 antibody on ice for 30 min, followed by incubation with anti-APC-conjugated magnetic beads. Monocytes were enriched on a magnetic separator. To test the purity of sorted monocytes, cells were stained with APC conjugated anti-CD14 antibody, followed by FACS examination. Cells with purity above 95% were used for subsequent experiments. Cells were resuspended in RPMI-1640 medium containing 10% (v/v) fetal bovine serum and 50 ng/ml human M-CSF, followed by growing at 37 °C with a 5% CO_2_ humidified atmosphere for 7 days.

All blood samples were collected from healthy volunteers with their written informed consent. Healthy volunteers needed to fill out a questionnaire (PARS-3) about whether they had been exercising recently, as well as the intensity and frequency of their exercise, in order to rate their exercise activity [46,47]. Those who scored above 30 points were considered the exercise group, while those who scored below 30 points were considered the sedentary group. Study was licensed by the Ethics Committee of Peking University Third Hospital (IRB00006761-M2024837).

### Pulmonary bronchitis inflammation score

To perform pathological statistical analysis on lymphocytes infiltrating around the pulmonary bronchi, the outer diameter of areas containing lymphocytes and bronchial tissue, as well as the inner diameter of individual bronchi were calculated. The ratio of the two was then calculated.

### Hematoxylin and Eosin staining

Lungs were fixed in 4% paraformaldehyde (PFA; Sigma-Aldrich) for 12 h. Fixed tissues were washed twice using 75% ethanol and embedded in paraffin, followed by sectioning and staining with hematoxylin and eosin according to standard laboratory procedures.

### Lac-Phe injection

Lac-Phe was dissolved in DMSO: Kolliphor EL: Saline in a volume ratio of 1:1:18. Mice were intraperitoneally injected with 5 μl/g Lac-Phe per day for seven consecutive days. Otherwise, mice were injected with vehicles.

### Mass spectrometry

Mass spectrometry was conducted in the Ultimate 3000 ultra-high performance liquid chromatograph and Q-Exactive HF combination quadrupole mass spectrometer. Briefly, samples dissolved in 100 μl of acetonitrile: water (1:1, v/v) were separated using a BEH amide chromatography column (inner diameter 100 × 2.1 mm, 1.7 μm) with an injection volume of 5 μl. Mobile phase A consists of 5 mM ammonium acetate aqueous solution and 5% acetonitrile, while mobile phase B is acetonitrile. The flow rate is 0.35 ml/min, with the following linear gradient: 0 min, 94% B; 1 min, 94% B; 12 min, 70% B; 12.1 min, 50% B; 15 min, 50% B; 16 min, 94% B; 25 min, 94% B.

Mass spectrometry data acquisition (DDA-MS) was performed in a Q-Exactive HF combination quadrupole mass spectrometer in either positive or negative ion mode. Each acquisition cycle includes one measurement scan (MS1 scan) with a resolution of 60000 and a quality range of 70–1050 m/z. Perform 10 MS/MS scans in HCD mode with a resolution of 30000, using 20, 40, and 60 stepwise collision energy normalization. The dynamic exclusion time is set to 10 seconds. For MS1 and MS/MS scans, the automatic gain control targets are set to 3 x 10^6^ (maximum sampling time of 100 ms) and 1 x 10^5^ (maximum sampling time of 80 ms), respectively. The ion source parameters are: positive ion mode spray voltage is 3.5 kV, negative ion mode spray voltage is 3.0 kV; ion source sheath gas = 40; Auxiliary gas = 10; Capillary temperature = 320 °C; Probe heater temperature = 350 °C; S-lens RF level = 60. Samples were analyzed in a random order, quality control (QC) samples were prepared by merging equal volume samples from all study instances, and every five samples were analyzed in the entire LC-MS analysis sequence.

### Plasmid transfection and lentiviral production

Transfection reagent JetPRIME (Polyplus Transfection) was used to conduct transfection in HEK293T cells. HEK293T cells were plated 14 h earlier to achieve a density of about 70% when transfection was performed. Lentiviral plasmids containing exogenous genes of interest and packaging plasmids pSPAX2 and pMD2.G were transfected into HEK293T cells at a ratio of 4:3:1. 48 h later, supernatants were collected, followed by centrifugation at 1,000 rpm for 5 min and filtration using 0.45 μm sterile syringe filters (Merck Millipore). The filtrate was then concentrated with a 100 kD Amicon ultra centrifugal filter unit (Merck Millipore). Macrophage cells were incubated with lentiviruses for 6 h, followed by culture medium replacement with fresh medium. 2 μg/ml puromycin was added to the medium 48 h post infection.

### Protein transfection using cell-penetrating peptides

For a single well of a 6-well plate, target peptides were incubated with CPP (1:1) in 100 μl of PBS at room temperature for 30 min, after which the peptide‒CPP mixture was added to the cell medium. The culture medium was replaced with fresh medium 3 h later. Commercial cell-penetrating peptides (ab142343) were purchased from Abcam.

### Cell viability and cytotoxicity assay

Cell pellets from cells stimulated with inflammasome activators were used to measure cell viability through checking cellular ATP levels with a CellTiter-Glo luminescent cell viability assay kit (Promega). The supernatants from cells stimulated with inflammasome activators were collected and subjected to lactate dehydrogenase (LDH) assay to assess cell death using a CytoTox 96 non-radioactive cytotoxicity assay kit (Promega). Both the supernatants and cell pellets were collected for immunoblotting analyses.

### ELISA

ELISA kits detecting human IL-1β (D711068), mouse IL-1β (D721017), mouse IL-6 (D721022) and mouse TNF-α (D721217) were purchased from BBI life sciences (Shanghai). Cell culture supernatants were collected and subjected to ELISA assays following manufacturer’s instructions.

### Recombinant protein expression and purification

For recombinant expression of GST-tagged proteins, cDNA of interest was cloned into pGEX-6P-1 vector. Plasmids were transformed into *E. coli* strain BL21 and the bacteria grew in LB medium supplemented with 100 μg/ml ampicillin at 37 °C for 8 h, followed by addition of IPTG to a final concentration of 0.5 mM and shaking at 150 rpm at 37 °C for 3 h. Cells were harvested and resuspended with PBS containing 1% Triton-X-100 and lysed by sonication. Cell lysates were centrifuged at 13,000 rpm at 4 °C for 30 min and filtered through sterile syringe filters. For crude purification of recombinant proteins, the filtered lysate was loaded on column containing glutathione sepharose 4B resin, followed by washing with PBS containing 1% Triton-X-100. The protein was eventually eluted by 1 ml 10 mM L-Glutathione solution. For removal of LPS contaminations, crudely purified proteins were further eluted through Pierce high-capacity endotoxin removal spin columns and heparin columns to remove LPS and bacterial genomic DNA, respectively. GST tags were removed by a PreScission protease.

### Immunoprecipitation

HEK293T cells were seeded on 6-well culture plates and were transfected with plasmids 16 h later. 24 h post transfection, cells were washed twice with PBS and lysed in a pre-chilled buffer containing 150 mM NaCl, 50 mM Tris (pH 7.5), 1 mM EDTA, 0.5% digitonin, 1% protease inhibitor cocktail and 10% glycerol on ice for 30 min. For immunoprecipitation using primary cells, cells post stimulation or treatments were lysed in a pre-chilled PBS buffer containing 0.5% digitonin, 1% protease inhibitor cocktail on ice for 30 min. Cell lysates were centrifuged at 13,000 rpm at 4 °C for 10 min. Primary antibodies and their isotype control IgG were immobilized to AminoLink Coupling Resin (ThermoFisher Scientific) following the manufacturer’s instructions. Supernatants were incubated with immobilized antibodies at 4 °C for 2 h. Resins were washed three times with PBS containing 0.5% digitonin, followed by immunoprecipitant detachment in 0.1 M Glycine-HCl, pH 2.7. Supernatants were neutralized by adding 1/10 the volume of 2 M Tris-HCl, pH 8.0, followed by immunoblotting with the indicated antibodies.

### Protein and small molecule docking

Protein docking was performed following previous methods [4]. Briefly, the protein structure of mouse CypD (aa 45–206, AF-Q99KR7-F1) and γ-Glu-Cys were used. The γ-Glu-Cys structure was downloaded from MolView. Protein structures were processed in Python molecule viewer and then docked using AutoDock of MGLtools version 1.57 following software instructions. Docked molecules were aligned with the CypD structures in PyMol version 2.4.0.

### Immunofluorescence

Cells adhered to covers precoated with poly-L-lysine were stimulated with inflammasome activators, followed by fixation with 4% paraformaldehyde for 10 min at room temperature. Cells were then washed twice with PBS and permeabilized with PBS containing 1% Triton-X-100 for 10 min, followed by washing with PBS twice and blocking in 10% normal goat serum at 37 °C for 30 min. Cells were incubated with primary antibody for 2 h, washed with PBS three times and further incubated with fluorescence-conjugated secondary antibody for 1 h. Nuclei were stained with DAPI. Anti-CNDP2 (ab241126) and anti-GSDMD (93709, CST) were used. For coimmunostaining using antibodies with the same origin, Lightning-Link kits (ab236553 and ab269900, Novus Biologicals) were used to directly link fluorescence to primary antibodies. Cells were visualized through an UltraVIEW VoX imaging system (PerkinElmer).

### Yeast two-hybrid screening

Yeast strain AH109 was inoculated into 5ml of YPDA medium and shaken at 250 rpm at 30 °C for 16 h. The overnight culture was transferred to a flask containing 50ml of YPDA and incubated at 30 °C for 3 h. Yeast cells were collected by centrifugation at 4000 rpm for 10 min and resuspended in 1XTE/LiAc buffer (pH 7.5) containing 0.1M lithium acetate, 0.1M Tris-HCl and 10mM EDTA. A mixture of plasmid DNA and carrier DNA was added to the yeast cell suspension and vortexed to mix well. PEG/LiAc buffer containing 40% polyethylene glycol 3350, 0.1M lithium acetate, 0.1M Tris-HCl and 10mM EDTA was then added to the suspension and vortexed at a high speed. The suspension was shaken at 200 rpm at 30 °C for 30 min, followed by the addition of DMSO and heat shock in a 42 °C water bath for 15 min. Cells were chilled on ice for 2 min and resuspended to plated on appropriate SD agar plates. A mouse bone marrow cDNA library was used for Y2H screening with Gal4 DNA binding domain (BD)-tagged CNDP2 as bait. BD-tagged baits and Gal4 DNA activating domain (AD)-tagged preys were co-transfected into yeast strain AH109, and double-positive clones were selected on SD medium lacking adenine, histidine, leucine and tryptophan.

### Statistical analysis

No statistical methods were used to predetermine sample size. Experiments were independently repeated at least three times to achieve statistical significance. No randomization or blinding procedures were used in this study. No samples were excluded from the analysis. Data were shown as means ± SD of three technical replicates. Data with normal distribution determined by Shapiro–Wilk normality test were statistically analyzed by two tailed Student’s t tests if not specified. The Gehan-Breslow-Wilcoxon test was used for the analysis of survival data. Data were analyzed by GraphPad Prism 9.0. *P*-values ≤ 0.05 were termed as significant; *P*-values > 0.05 were termed as non-significant.

## Supporting information

S1 Fig CNDP2 is essential for enhancing immune responses during exercise. (A–D) Wild-type mice were intraperitoneally injected with 5 μl/g Lac-Phe per day for seven consecutive days. Mice were then intratracheally inoculated with 3 x 10^4^ CFU of *K. pneumoniae* (*K. p.*). Serum levels of IL-1β (A), IL-6 (B) and TNF-α (C) were determined through ELISA assays 24 h post infection. Bacterial load was examined in mouse lung tissues post infection (D). (E) Peritoneal macrophages from *Cndp2*^flox/flox^ and *Cndp2*^flox/flox^;*Lyz2*-Cre mice were collected and immunoblotted with antibodies against the indicated proteins. (F–K) *Cndp2*^flox/flox^ and *Cndp2*^flox/flox^;*Lyz2*-Cre mice were housed under a sedentary state or exercised for one month, followed by intratracheal inoculation of 3 x 10^4^ CFU of *K. pneumoniae*. Serum levels of IL-1β (F), IL-6 (G) and TNF-α (H) were determined through ELISA assays 24 h post infection. Mouse lungs were fixed and subjected to Hematoxylin and Eosin staining 24 h post infection (I). Inflammation scores were calculated through comparing degree of lymphocyte infiltration around bronchus between distinct conditions (J). Survivals of mice were monitored (K). For K, n = 11. Scale bar: 100 μm. Data were shown as means±SD. Experiments were repeated three times with similar results.
(TIFF)

S2 Fig CNDP2 controls inflammasome hyperactivation post exercise. (A, B) Wild-type mice were housed under a sedentary state or exercised for one month. Peritoneal macrophages were collected from these mice and cultured in the presence of *K. pneumoniae* at an MOI of 10 for 1 h. PI uptake (A) and IL-1β release (B) were measured over time. (C–G) Wild-type mice were housed under a sedentary state or exercised for one month. BMDM cells were collected from these mice and cultured in the presence of *K. pneumoniae* at an MOI of 10 for 1 h. Cells were then supplemented with fresh medium containing 100 μg/ml gentamycin. Cells were collected and immunoblotted with antibodies against the indicated proteins 6 h post infection (C). Band intensities of cleaved caspase-1 (p20) and GSDMD (GSDMD-N) were calculated and shown as relative to that of β-actin (D). Cell culture supernatants were collected for ELISA assays of IL-1β 24 h post infection (E). Percentages of viable cells were determined by examining cellular ATP levels in cell pellets 24 h post infection (F). Percentages of cells undergoing pyroptosis were calculated by assessing released LDH in cell culture supernatants 24 h post infection (G). (H, I) *Cndp2*^+/+^ and *Cndp2*^–/–^ mice were housed under a sedentary state or exercised for one month. BMDM cells were collected from these mice and cultured in the presence of *K. pneumoniae* at an MOI of 10 for 1 h. Cells were then supplemented with fresh medium containing 100 μg/ml gentamycin. Cells were collected and immunoblotted with antibodies against the indicated proteins 6 h post infection (H). Band intensities of cleaved caspase-1 (p20) and GSDMD (GSDMD-N) were calculated and shown as relative to that of β-actin (I). (J–N) *Nlrp3*^+/+^ and *Nlrp3*^–/–^ mice were housed under a sedentary state or exercised for one month. Peritoneal macrophages were collected from these mice and cultured in the presence of *K. pneumoniae* at an MOI of 10 for 1 h. Cells were then supplemented with fresh medium containing 100 μg/ml gentamycin. Cells were collected and immunoblotted with antibodies against the indicated proteins 6 h post infection (J). Band intensities of cleaved caspase-1 (p20) and GSDMD (GSDMD-N) were calculated and shown as relative to that of β-actin (K). Cell culture supernatants were collected for ELISA assays of IL-1β 24 h post infection (L). Percentages of viable cells were determined by examining cellular ATP levels in cell pellets 24 h post infection (M). Percentages of cells undergoing pyroptosis were calculated by assessing released LDH in cell culture supernatants 24 h post infection (N). Data were shown as means±SD. Experiments were repeated three times with similar results.
(TIFF)

S3 Fig CNDP2 binds the N-terminus of GSDMD. (A) Plasmids encoding CNDP2 and GSDMD were co-transfected into HEK293T cells for 24 h, followed by immunoprecipitation with a control IgG or antibody against FLAG. Precipitates were immunoblotted with antibodies against the indicated proteins. (B) Wild-type BMDM cells were immunoprecipitated with a control IgG or antibody against CNDP2. Precipitates were immunoblotted with antibodies against the indicated proteins. (C, D) Peritoneal macrophages were collected from wild-type mice and cultured in the presence of *K. pneumoniae* at an MOI of 10 for 1 h. Cells were then supplemented with fresh medium containing 100 μg/ml gentamycin for 6 h. Cells were collected and immunoprecipitated with a control IgG or antibody against CNDP2. Precipitates were immunoblotted with antibodies against the indicated proteins (C). Band intensities of precipitated GSDMD were relative to that of precipitated CNDP2 (D). (E) A scheme of GSDMD truncations was shown. (F) A scheme of CNDP2 truncations was shown. (G) Plasmids encoding CNDP2 and GSDMD truncations were co-transfected into HEK293T cells for 24 h. Cells were then lysed and immunoprecipitated with a control IgG or antibody against CNDP2. Precipitates were immunoblotted with antibodies against the indicated proteins. (H) Plasmids encoding GSDMD and CNDP2 truncations were co-transfected into HEK293T cells for 24 h. Cells were then lysed and immunoprecipitated with a control IgG or antibody against FLAG. Precipitates were immunoblotted with antibodies against the indicated proteins. (I, J) Recombinant caspase-1 subunits p20 and p10 were incubated with GSDMD with or without the presence of CNDP2 for 1 h. Protein mixtures were immunoblotted with antibodies against the indicated proteins (I). Band intensities of cleaved GSDMD (GSDMD-N) were relative to that of p20 (J). (K) Plasmids encoding CNDP2 and caspase-1 were co-transfected into HEK293T cells for 24 h, followed by immunoprecipitation with a control IgG or antibody against CNDP2. Precipitates were immunoblotted with antibodies against the indicated proteins. (L–P) *Cndp2*^–/–^ BMDM cells were transfected with or without 10 mM Cys-Gly through cell-penetrating peptides for 6 h. Cells were then cultured in the presence of *K. pneumoniae* at an MOI of 10 for 1 h. Cells were supplemented with fresh medium containing 100 μg/ml gentamycin. Cells were collected and immunoblotted with antibodies against the indicated proteins 6 h post infection (L). Band intensities of cleaved caspase-1 (p20) and GSDMD (GSDMD-N) were calculated and shown as relative to that of β-actin (M). Cell culture supernatants were collected for ELISA assays of IL-1β 24 h post infection (N). Percentages of viable cells were determined by examining cellular ATP levels in cell pellets 24 h post infection (O). Percentages of cells undergoing pyroptosis were calculated by assessing released LDH in cell culture supernatants 24 h post infection (P). Data were shown as means±SD. Experiments were repeated three times with similar results.
(TIFF)

S4 Fig γ-Glu-Cys promotes the establishment of inflammasome hyperactivation. (A, B) Glutathione Sepharose 4B resin was incubated with CNDP2 and GSDMD N-terminus for 2 h, followed by further incubation with GST proteins for 2 h and a pull-down assay. Precipitates and input proteins were immunoblotted with antibodies against the indicated proteins (A). Band intensities of pull-down GST were calculated and shown as relative to that of input GST (B). (C, D) Glutathione Sepharose 4B resin was incubated with wild-type CNDP2 or mutant (D132A) with the presence of GSDMD N-terminus for 2 h, followed by further incubation with GST proteins for 2 h and a pull-down assay. Precipitates and input proteins were immunoblotted with antibodies against the indicated proteins (C). Band intensities of pull-down GST were calculated and shown as relative to that of input GST (D). (E–I) Peritoneal macrophages were collected from *Cndp2*^+/+^ and *Cndp2*^–/–^ mice and transfected with 10 mM γ-Glu-Cys with the help of cell-penetrating peptides for 6 h. Cells were further cultured in the presence of *K. pneumoniae* at an MOI of 10 for 1 h. Cells were then supplemented with fresh medium containing 100 μg/ml gentamycin. Cells were collected and immunoblotted with antibodies against the indicated proteins 6 h post infection (E). Band intensities of cleaved caspase-1 (p20) and GSDMD (GSDMD-N) were calculated and shown as relative to that of β-actin (F). Cell culture supernatants were collected for ELISA assays of IL-1β 24 h post infection (G). Percentages of viable cells were determined by examining cellular ATP levels in cell pellets 24 h post infection (H). Percentages of cells undergoing pyroptosis were calculated by assessing released LDH in cell culture supernatants 24 h post infection (I). (J–L) Wild-type mice were housed under a sedentary state or exercised for one month. Mice were then intratracheally inoculated with 3 x 10^4^ CFU of *K. pneumoniae*. Plasma levels of GSH in blood (J) and intracellular levels of GSH in peritoneal macrophages (K) were determined through quantitative mass spectrometry. Intracellular levels of γ-Glu-Cys in peritoneal macrophages (L) were determined through quantitative mass spectrometry. (M) Wild-type mice were housed under a sedentary state or exercised for one month. Peritoneal macrophages collected from these mice and cultured in the presence of *K. pneumoniae* at an MOI of 10 with or without bestatin for 1 h. Intracellular levels of γ-Glu-Cys were determined through quantitative mass spectrometry. (N, O) *Cndp2*^+/+^ and *Cndp2*^–/–^ BMDM cells were incubated with 10 mM γ-Glu-Cys in DMEM media for 6 h. Cells were further cultured in the presence of *K. pneumoniae* at an MOI of 10 for 1 h. Cells were then supplemented with fresh medium containing 100 mg/ml gentamycin. Cells were collected and subjected to examination of intracellular levels of γ-Glu-Cys (N) or GSH (O) through quantitative mass spectrometry. Data were shown as means±SD. Experiments were repeated three times with similar results.
(TIFF)

S5 Fig γ-Glu-Cys protects mitochondrial membrane integrity. (A–E) *Cndp2*^+/+^ and *Cndp2*^–/–^ BMDM cells were collected from these mice and incubated with 10 mM γ-Glu-Cys in DMEM media for 6 h. Cells were further cultured in the presence of *K. pneumoniae* at an MOI of 10 for 1 h. Cells were then supplemented with fresh medium containing 100 μg/ml gentamycin. Cells were collected and immunoblotted with antibodies against the indicated proteins 6 h post infection (A). Band intensities of cleaved caspase-1 (p20) and GSDMD (GSDMD-N) were calculated and shown as relative to that of β-actin (B). Cell culture supernatants were collected for ELISA assays of IL-1β 24 h post infection (C). Percentages of viable cells were determined by examining cellular ATP levels in cell pellets 24 h post infection (D). Percentages of cells undergoing pyroptosis were calculated by assessing released LDH in cell culture supernatants 24 h post infection (E). (F) Glutathione Sepharose 4B resin was incubated with CNDP2 and GSDMD N-terminus for 2 h to generate resin-linked γ-Glu-Cys. Resins were incubated with HEK293T cell lysates expressing FLAG-tagged proteins of interest, followed by ELISA assays for FLAG signals. (G) *Ppif*^+/+^ and *Ppif*^–/–^ mice were housed under a sedentary state or exercised for one month, followed by intratracheal inoculation of 3 x 10^4^ CFU of *K. pneumoniae*. Serum levels of IL-1β were determined through ELISA assays 24 h post infection. (H) *Ppif*^+/+^ and *Ppif*^–/–^ mice were housed under a sedentary state or exercised for one month, followed by intratracheal inoculation of 3 x 10^4^ CFU of *K. pneumoniae*. Intracellular levels of γ-Glu-Cys in peritoneal macrophages were determined through quantitative mass spectrometry 24 h post infection. (I–K) *Ppif*^+/+^ and *Ppif*^–/–^ mice were housed under a sedentary state or exercised for one month. Peritoneal macrophages were collected from these mice and cultured in the presence of *K. pneumoniae* at an MOI of 10 for 1 h. Cells were then supplemented with fresh medium containing 100 μg/ml gentamycin. Cell culture supernatants were collected for ELISA assays of IL-1β 24 h post infection (I). Percentages of viable cells were determined by examining cellular ATP levels in cell pellets 24 h post infection (J). Percentages of cells undergoing pyroptosis were calculated by assessing released LDH in cell culture supernatants 24 h post infection (K). Data were shown as means±SD. Experiments were repeated three times with similar results.
(TIFF)

S6 Fig γ-Glu-Cys inhibits the binding of GSDMD-N and CypD. (A) Plasmids encoding CypD and GSDMD were co-transfected into HEK293T cells for 24 h, followed by immunoprecipitation with a control IgG or antibody against FLAG. Precipitates were immunoblotted with antibodies against the indicated proteins. (B) A scheme for CypD truncations was shown. (C) Plasmids encoding CypD and GSDMD truncations were co-transfected into HEK293T cells for 24 h, followed by immunoprecipitation with a control IgG or antibody against FLAG. Precipitates were immunoblotted with antibodies against the indicated proteins. (D) Plasmids encoding GSDMD N-terminus and CypD truncations were co-transfected into HEK293T cells for 16 h, followed by immunoprecipitation with a control IgG or antibody against FLAG. Precipitates were immunoblotted with antibodies against the indicated proteins. (E) Structure of CypD (aa 45–206, AF-Q99KR7-F1) was docked with γ-Glu-Cys (left panel). Key residues of CypD interacting with γ-Glu-Cys were shown (right panel). (F) Biotin or biotin-labelled γ-Glu-Cys were incubated with wild-type or mutant CypD at 4 °C for 2 h, followed by a pulldown assay. Precipitates were immunoblotted. (G) BMDM cells collected from *Ppif*^+/+^ and *Ppif*^–/–^ mice were cultured in the presence of *K. pneumoniae* at an MOI of 10 for 1 h. Cells were then supplemented with fresh medium containing 100 mg/ml gentamycin. Cells were collected and subjected to cytosol/mitochondrion fractionation, followed by quantitative PCR analysis of cytoplasmic mitochondrial DNA 6 h post infection. (H) *Ppif*^–/–^ BMDM cells were infected with lentiviruses carrying full-length CypD and truncations for 48 h. Cells were further cultured in the presence of *K. pneumoniae* at an MOI of 10 for 1 h. Cells were then supplemented with fresh medium containing 100 μg/ml gentamycin. Cells were collected and subjected to cytosol/mitochondrion fractionation, followed by quantitative PCR analysis of cytoplasmic mitochondrial DNA 6 h post infection. (I) *Ppif*^+/+^ and *Ppif*^–/–^ BMDM cells were incubated with 10 mM γ-Glu-Cys for 6 h. Cells were further cultured in the presence of *K. pneumoniae* at an MOI of 10 for 1 h. Cells were then supplemented with fresh medium containing 100 μg/ml gentamycin. Cells were collected and subjected to cytosol/mitochondrion fractionation, followed by quantitative PCR analysis of cytoplasmic mitochondrial DNA 6 h post infection. Data were shown as means±SD. Experiments were repeated three times with similar results.
(TIFF)

S7 Fig γ-Glu-Cys enhances the anti-infection activity. (A–E) *Ppif*^+/+^ and *Ppif*^–/–^ mice were orally administered with 500 mg/kg γ-Glu-Cys per day for five consecutive days, followed by intratracheal inoculation of 3 x 10^4^ CFU of *K. pneumoniae*. Serum levels of IL-1β were determined through ELISA assays 24 h post infection (A). Mouse lungs were fixed and subjected to Hematoxylin and Eosin staining 24 h post infection (B). Inflammation scores were calculated through comparing degree of lymphocyte infiltration around bronchus between distinct conditions (C). Bacterial load was examined in mouse lung tissues post infection (D). Survivals of mice were monitored (E). (F–H) *Ppif*^+/+^ and *Ppif*^–/–^ BMDM cells were incubated with 10 mM γ-Glu-Cys for 6 h. Cells were further cultured in the presence of *K. pneumoniae* at an MOI of 10 for 1 h. Cells were then supplemented with fresh medium containing 100 μg/ml gentamycin. Cell culture supernatants were collected for ELISA assays of IL-1β 24 h post infection (F). Percentages of viable cells were determined by examining cellular ATP levels in cell pellets 24 h post infection (G). Percentages of cells undergoing pyroptosis were calculated by assessing released LDH in cell culture supernatants 24 h post infection (H). (I, J) *Cndp2*^+/+^ and *Cndp2*^–/–^ mice were orally administered with 500 mg/kg γ-Glu-Cys per day for five consecutive days, followed by intratracheal inoculation of 3 x 10^4^ CFU of *K. pneumoniae*. Bacterial load was examined in mouse lung tissues post infection (I). Intracellular levels of γ-Glu-Cys in peritoneal macrophages were determined through quantitative mass spectrometry (J). (K) Human monocytes were isolated from bloods of healthy volunteers undergoing exercise or sedentary during the past month. Cells were further cultured in the presence of *K. pneumoniae* at an MOI of 10 for 1 h. Cells were then supplemented with fresh medium containing 100 μg/ml gentamycin. Percentages of cells undergoing pyroptosis were calculated by assessing released LDH in cell culture supernatants 24 h post infection. (L) Human monocytes were isolated from bloods of healthy volunteers undergoing exercise or sedentary during the past month. Cells were further cultured in the presence of *K. pneumoniae* at an MOI of 10 with or without bestatin for 1 h. Cells were then supplemented with fresh medium containing 100 μg/ml gentamycin. Intracellular bacterial load was examined post infection. For E, n = 12; for K, n = 29. Scale bar: 100 μm. Data were shown as means±SD. Experiments were repeated three times with similar results.
(TIFF)

S8 Fig A CNDP2 metabolite promotes inflammasome hyperactivation and anti-infection activity. Exercise promotes the binding between CNDP2 and GSDMD in macrophages, leading to the generation of γ-Glu-Cys. During inflammasome activation, γ-Glu-Cys binds to mitochondrial CypD and prevents it from interacting with the GSDMD N-terminus, resulting in a controlled, limited release of mitochondrial DNA. This controlled mitochondrial signal shifts the response into a hyperactivation-like state.
(TIFF)

S1 Raw Gel. Raw images for the figures. Uncropped gels for Figures and Supplementary Figures.
(PDF)

S1 Data Source data and statistical analyses. (XLSX)
