# Supplementary material for: Exercise promotes host anti-infection activity through CNDP2 metabolite-mediated inflammasome hyperactivation
Source: PLoS Pathog. 2026 Oct 9;22(10):e1014554. doi: 10.1371/journal.ppat.1014554 (PMC13652265; doi:10.1371/journal.ppat.1014554)

Uncropped Gels for Figure 2

Fig. 2F

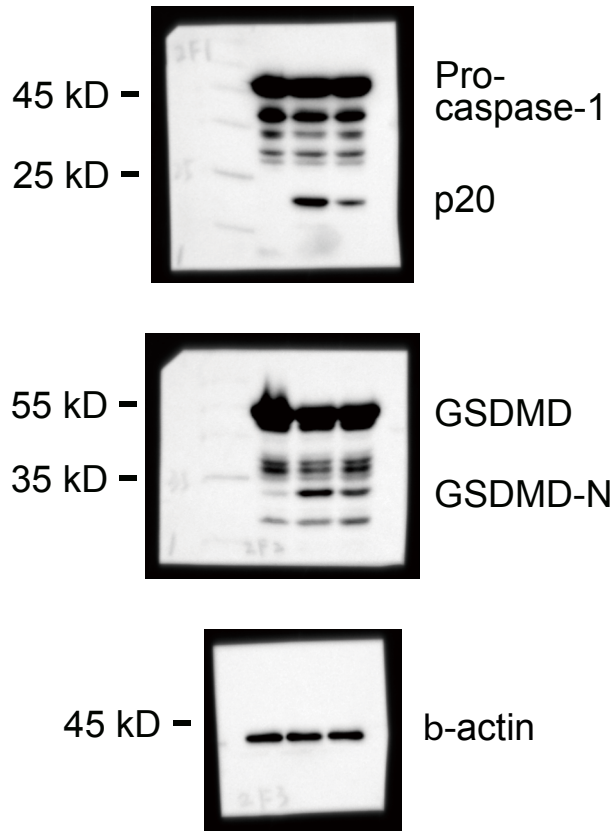

Fig.2H

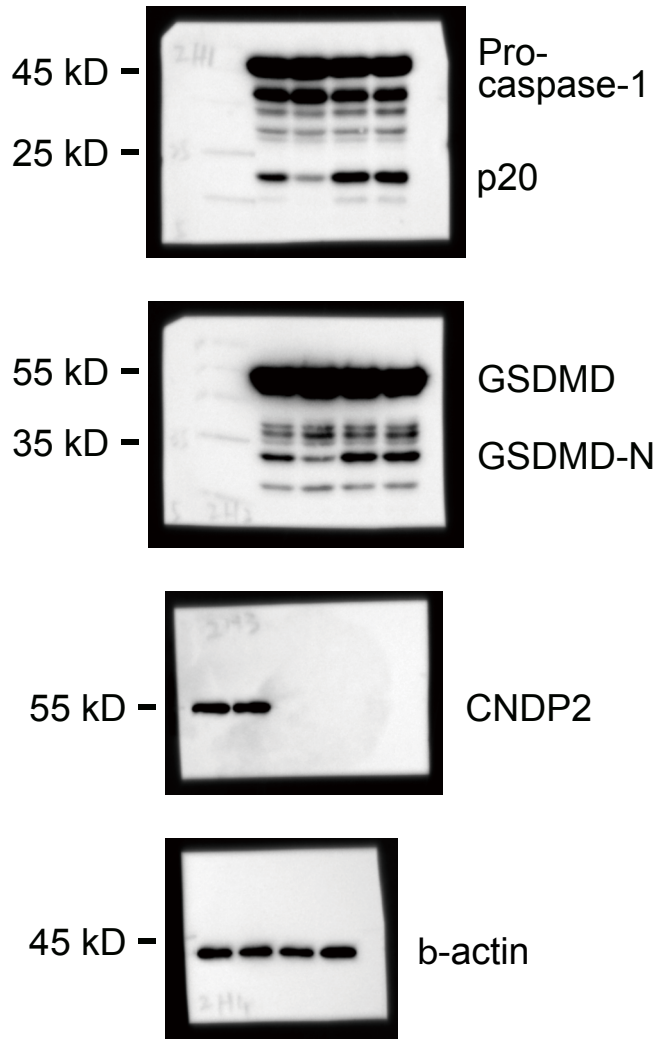

Uncropped Gels for Figure 3

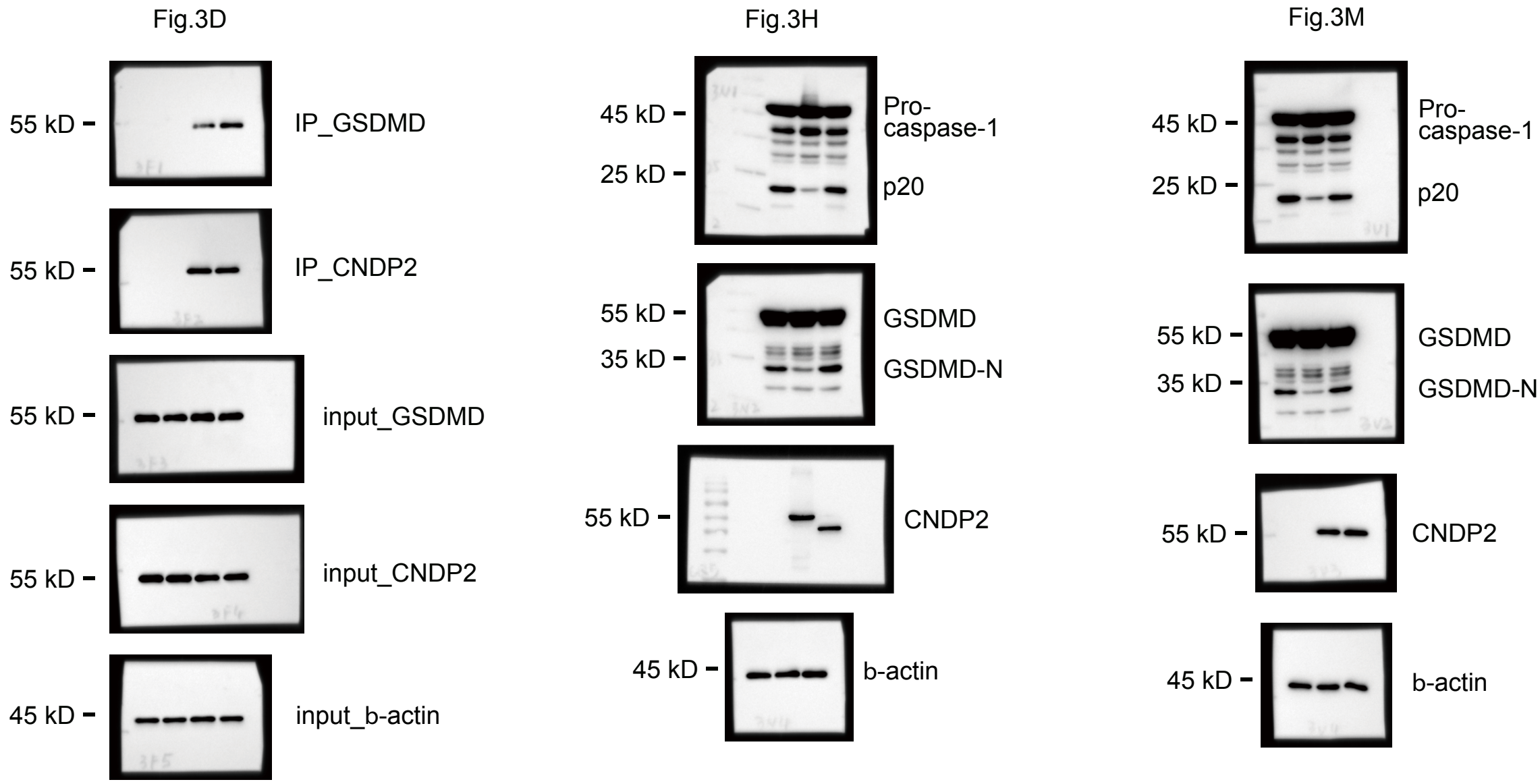

Uncropped Gels for Figure 4

Fig.4C

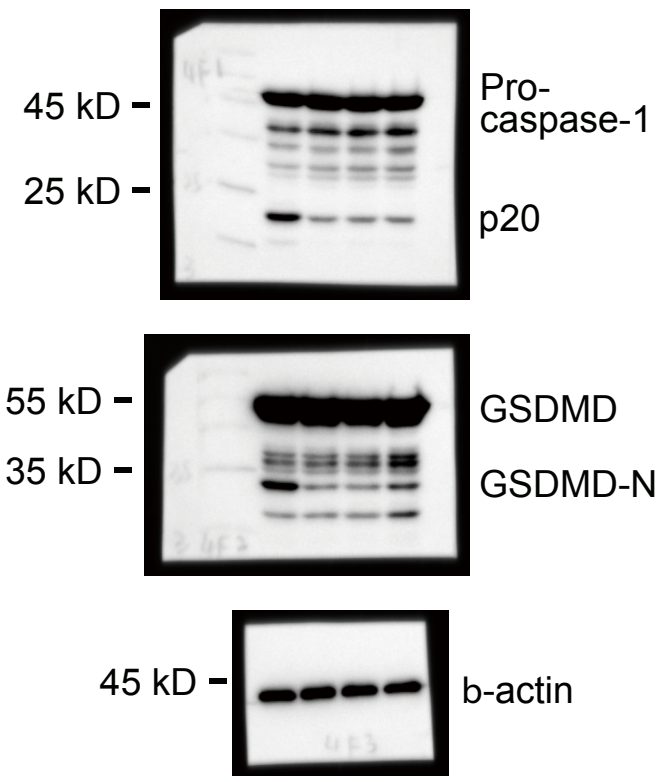

Fig.4H

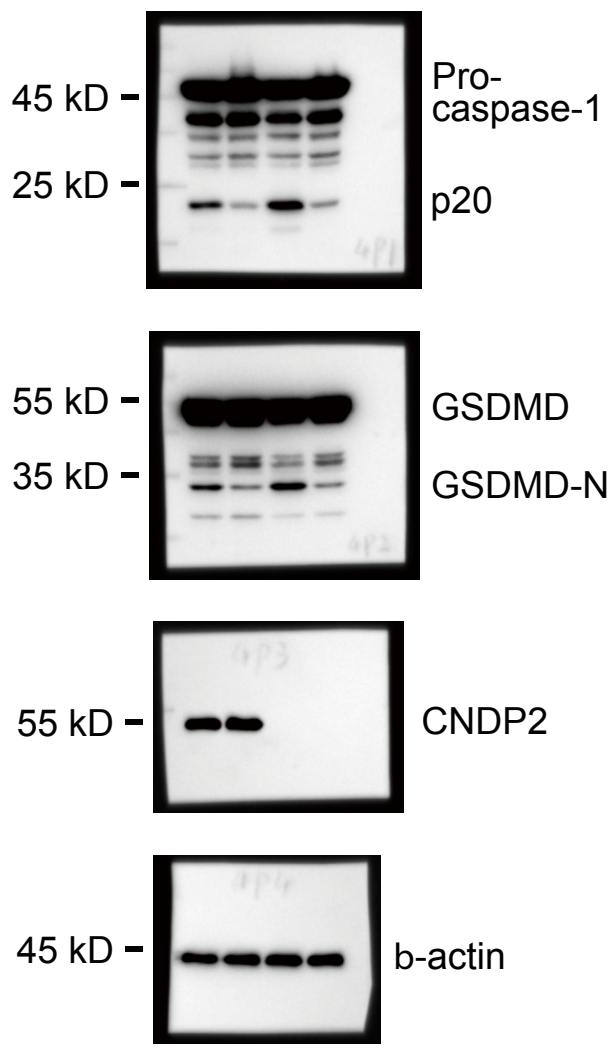

Uncropped Gels for Figure 5

Fig.5H

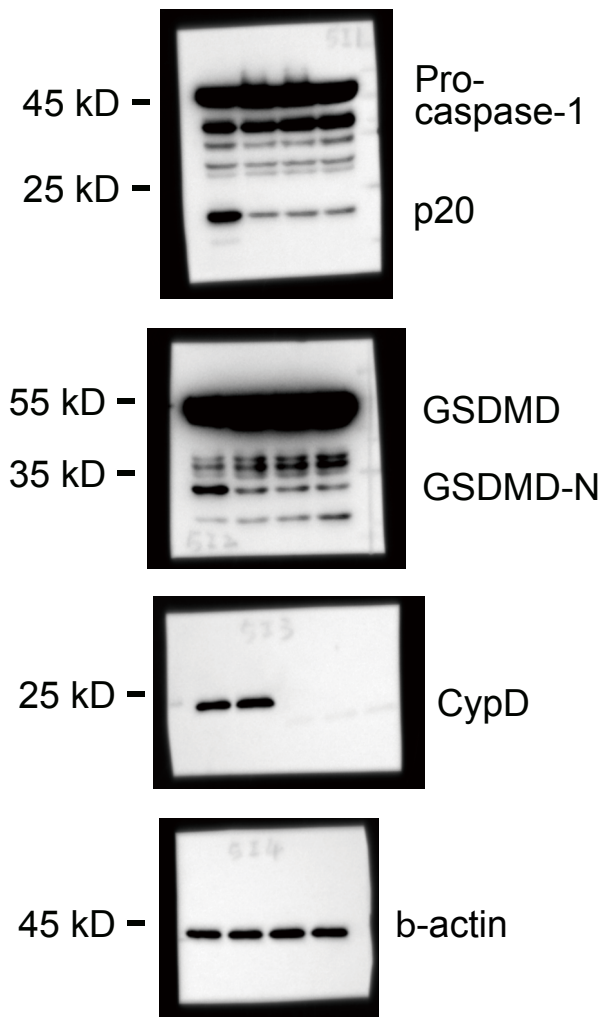

Fig.5K

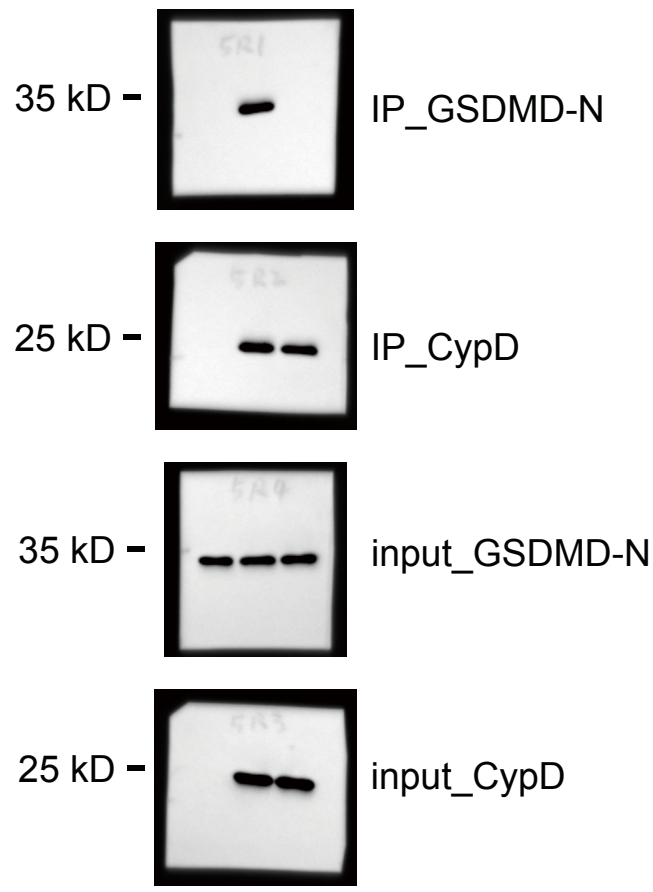

Fig.5L

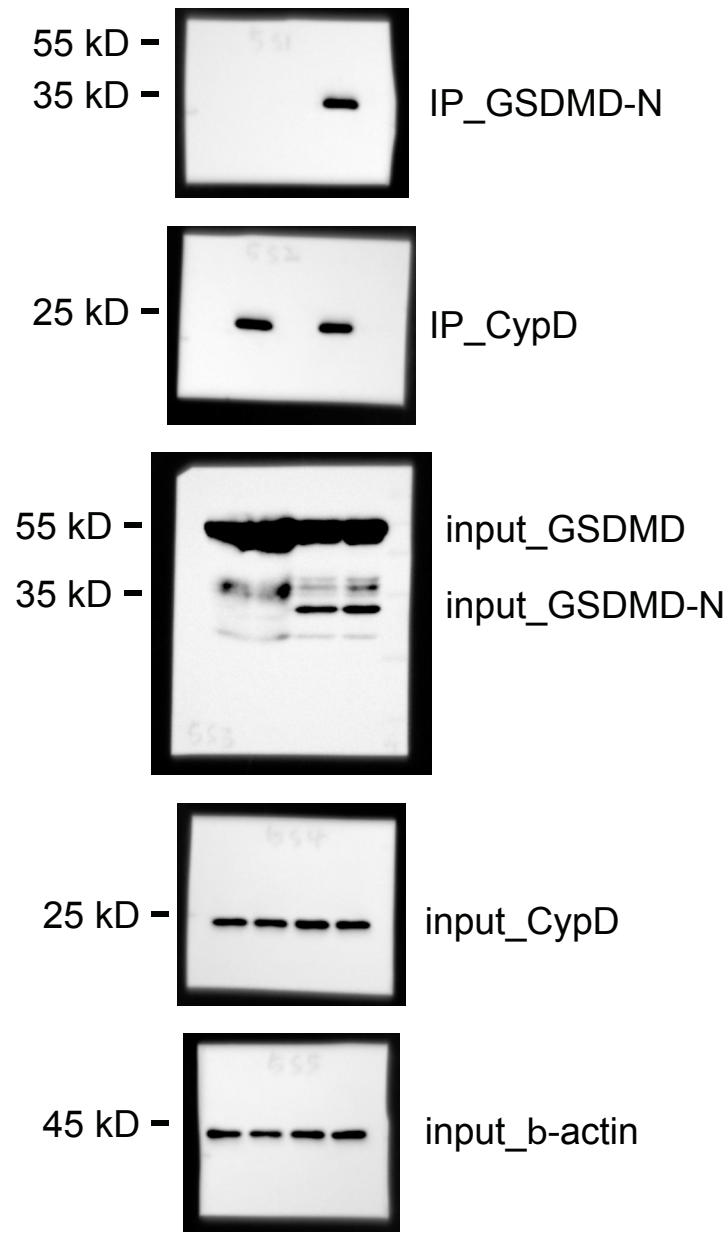

Fig.5M

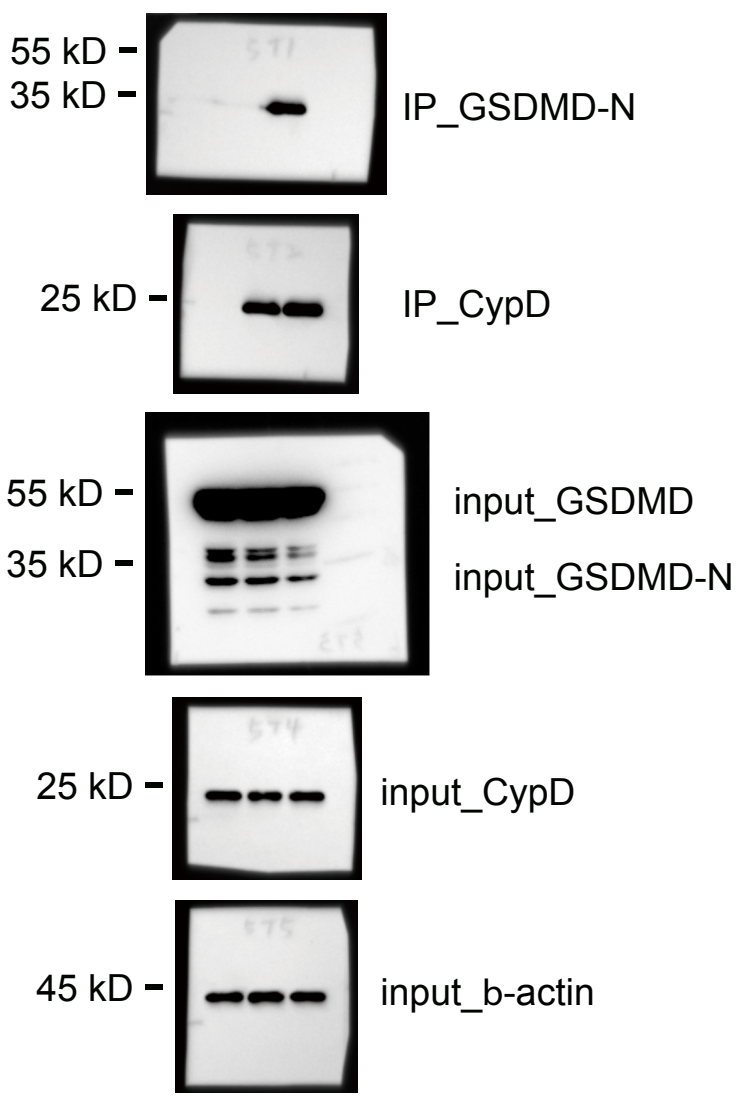

Uncropped Gels for Figure 6

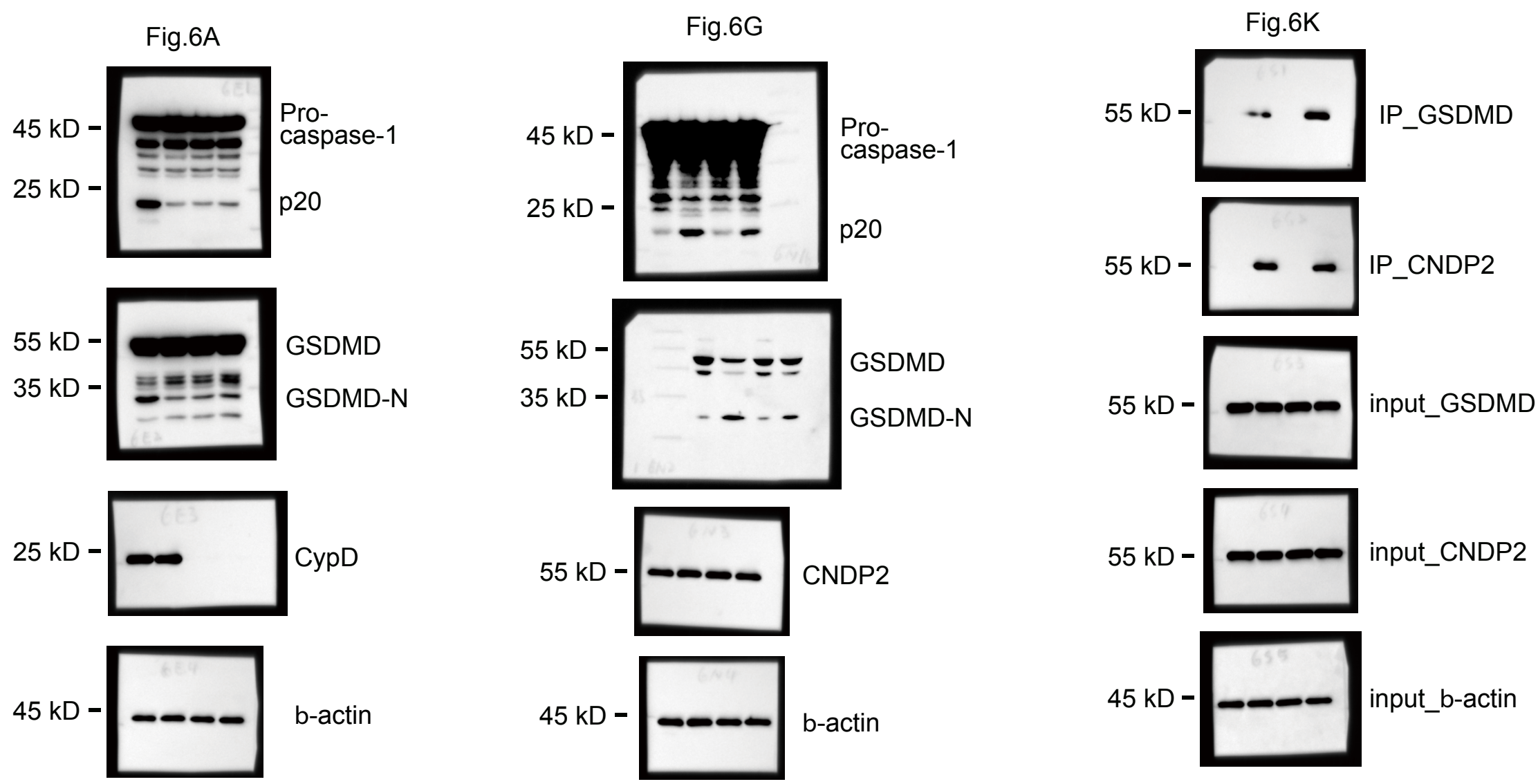

Uncropped Gels for Supplementary Figure 1

Fig.S1E

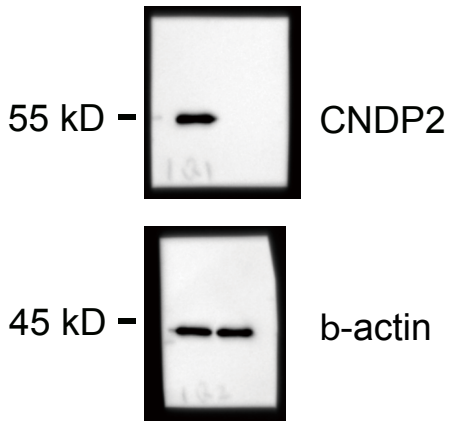

Uncropped Gels for Supplementary Figure 2

Fig.S2C

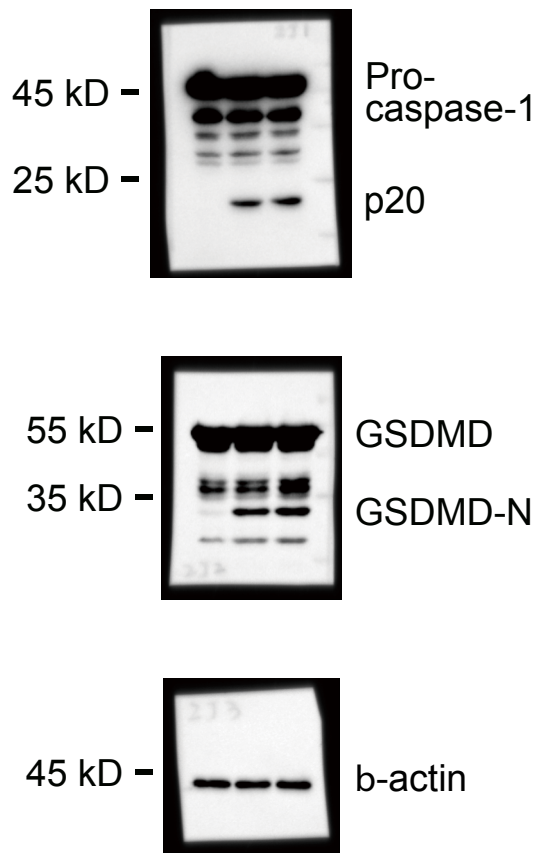

Fig.S2H

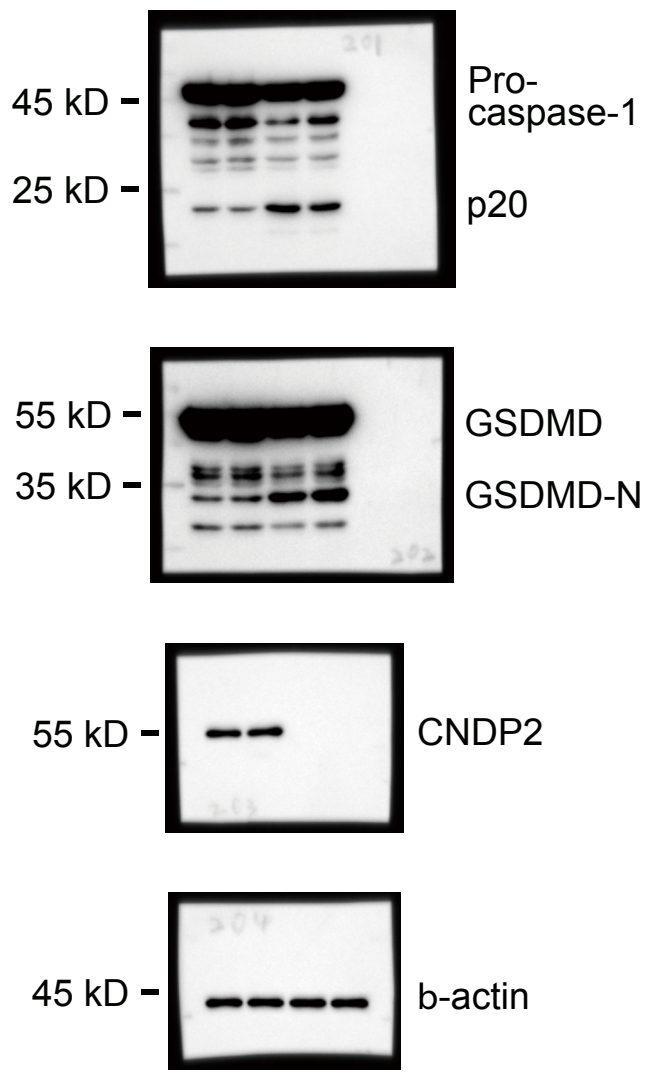

Fig.S2J

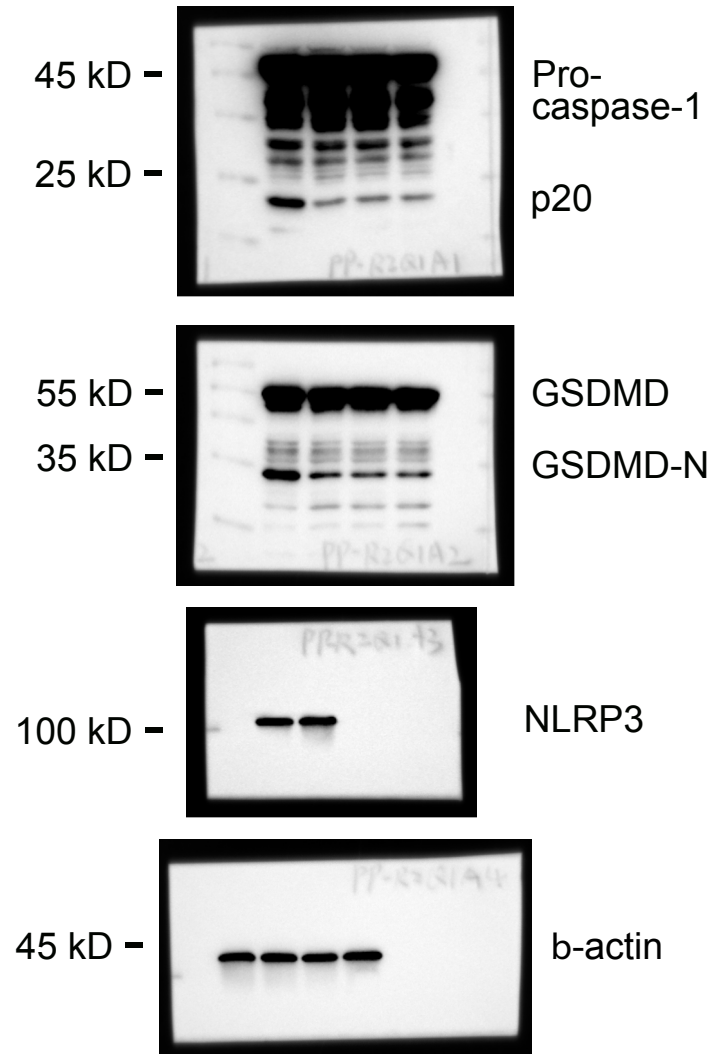

Uncropped Gels for Supplementary Figure 3

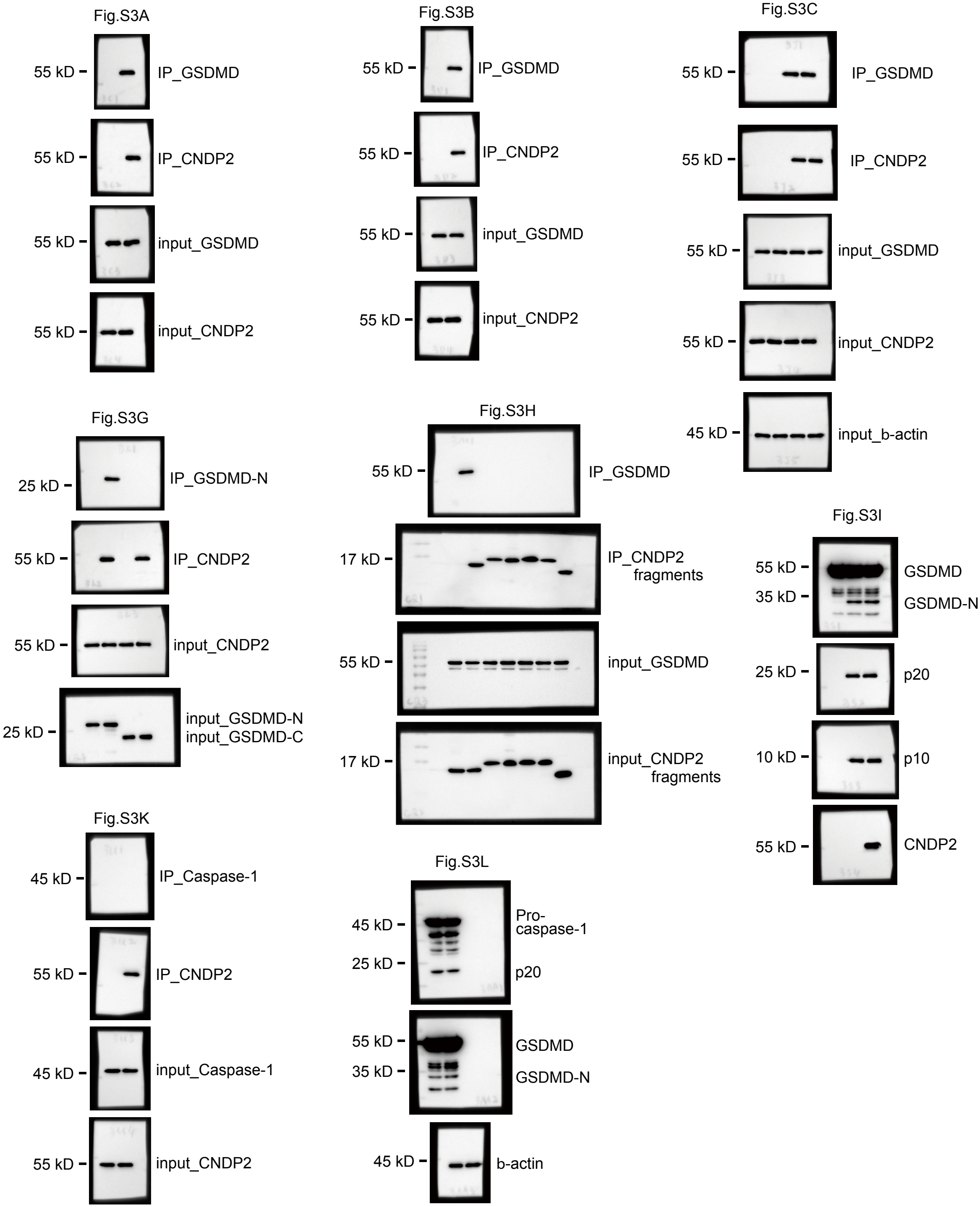

Uncropped Gels for Supplementary Figure 4

Fig.S4A

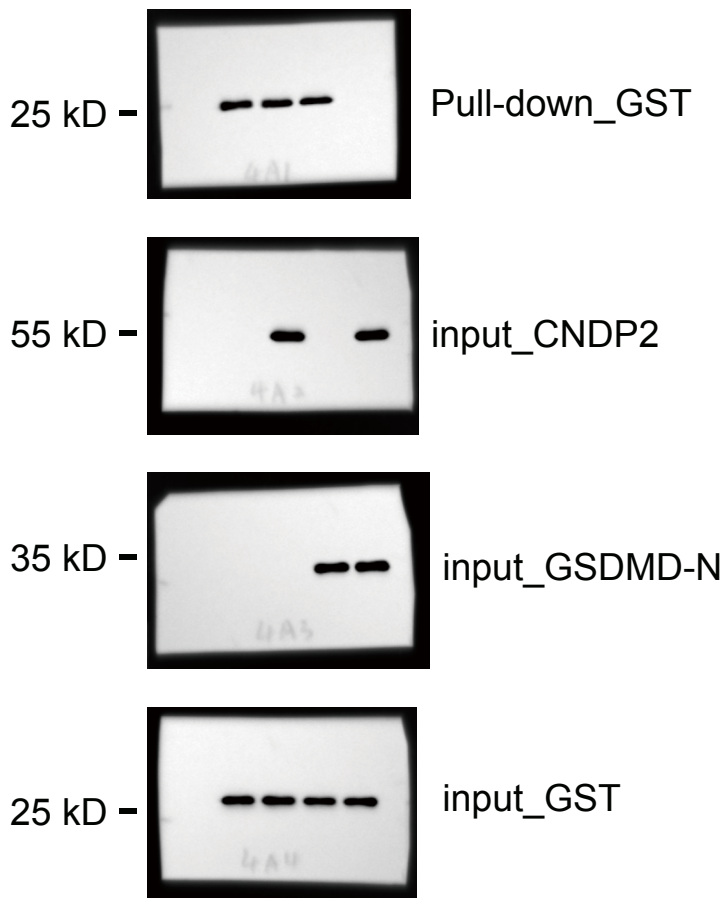

Fig.S4C

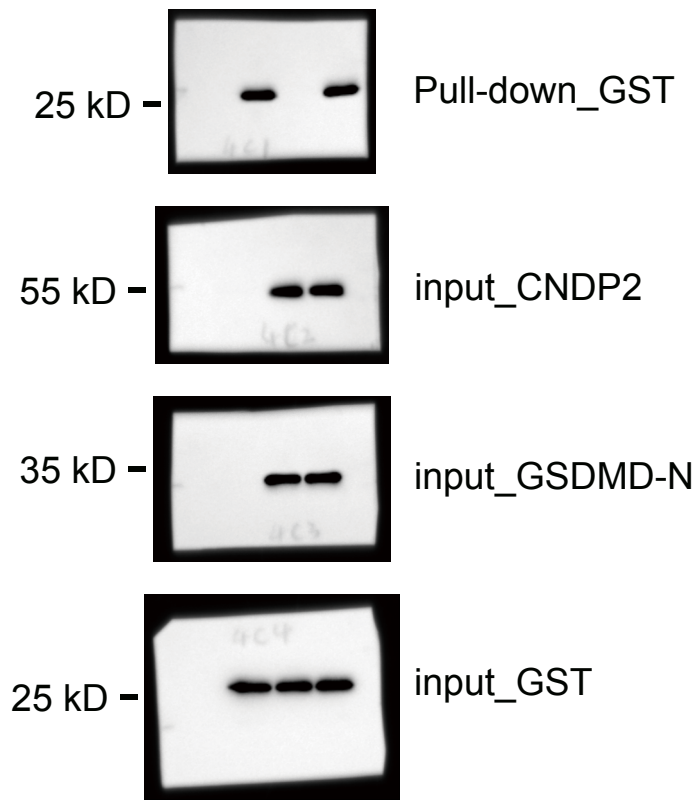

Fig.S4E

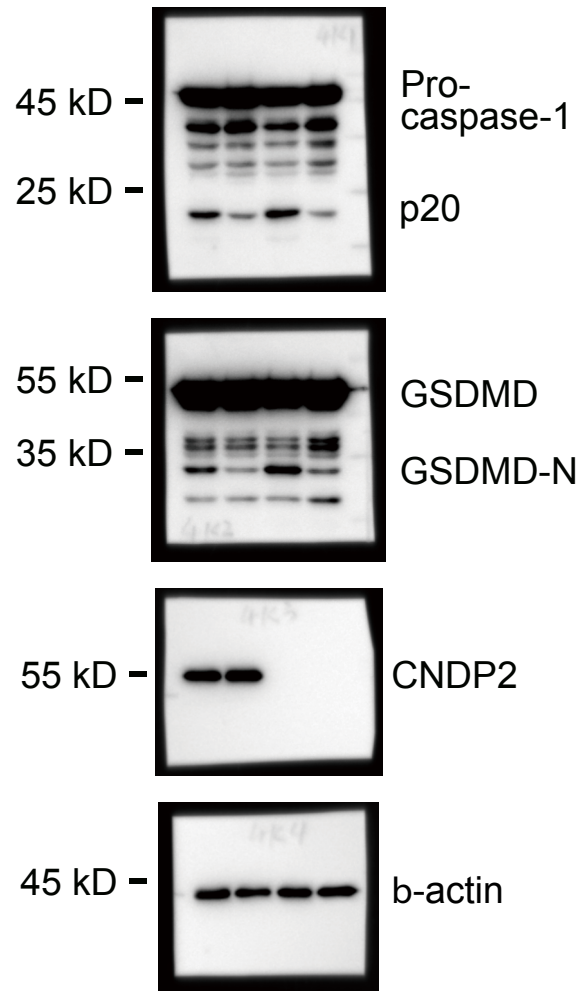

Uncropped Gels for Supplementary Figure 5

Fig.S5A

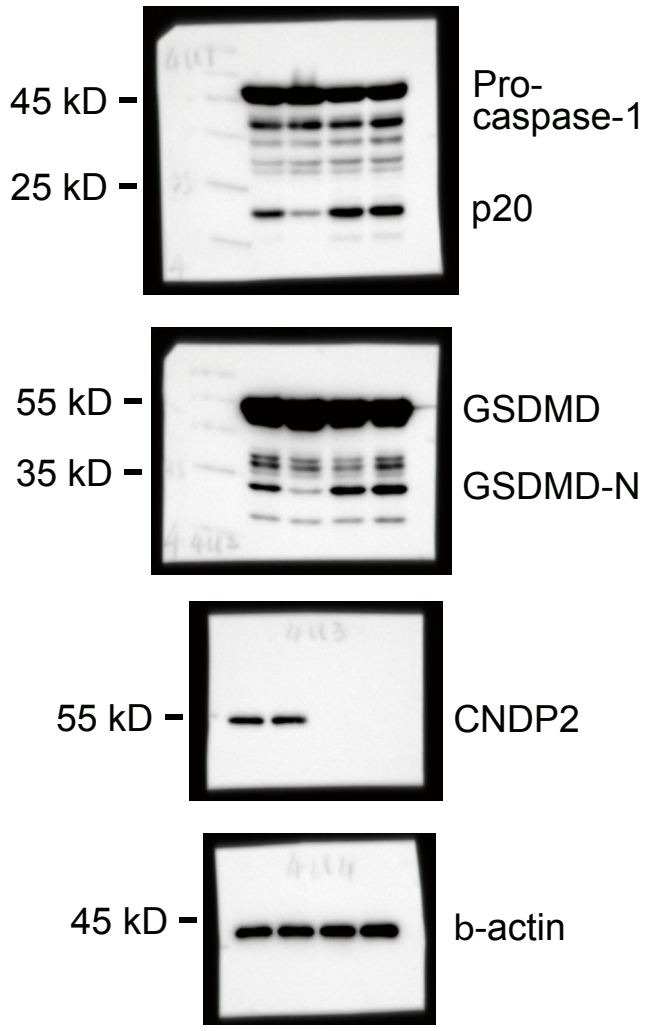

Uncropped Gels for Supplementary Figure 6

Fig.S6A

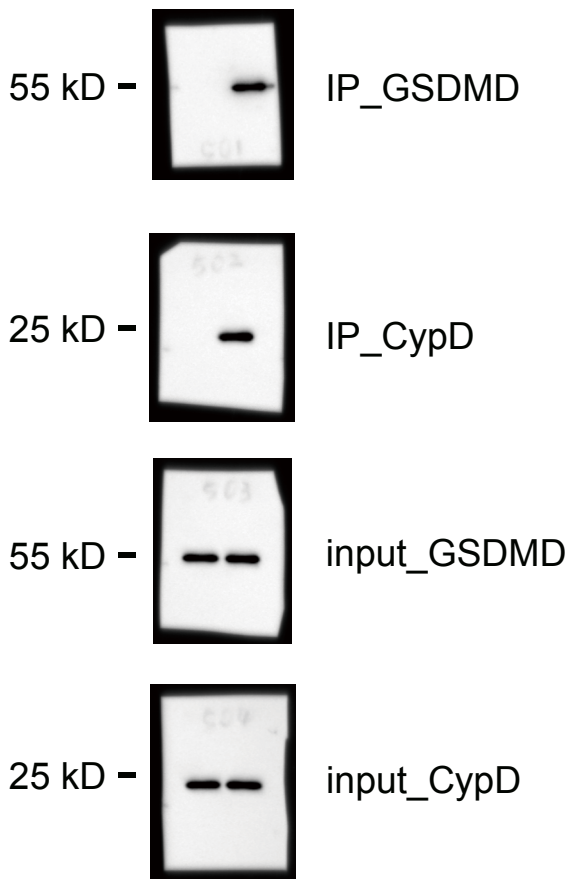

Fig.S6C

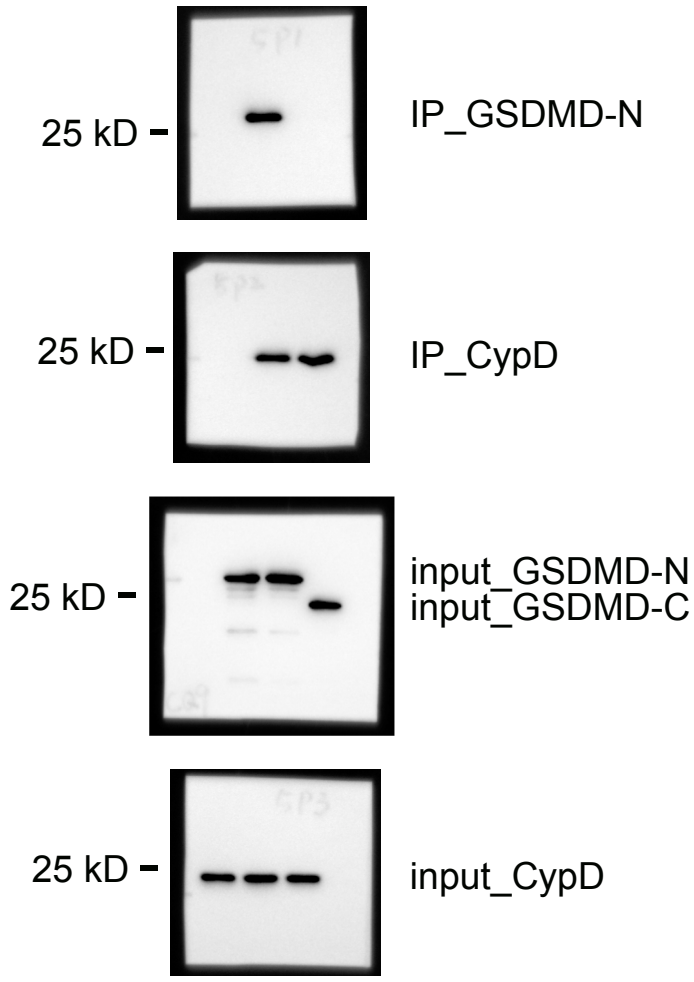

Fig.S6D

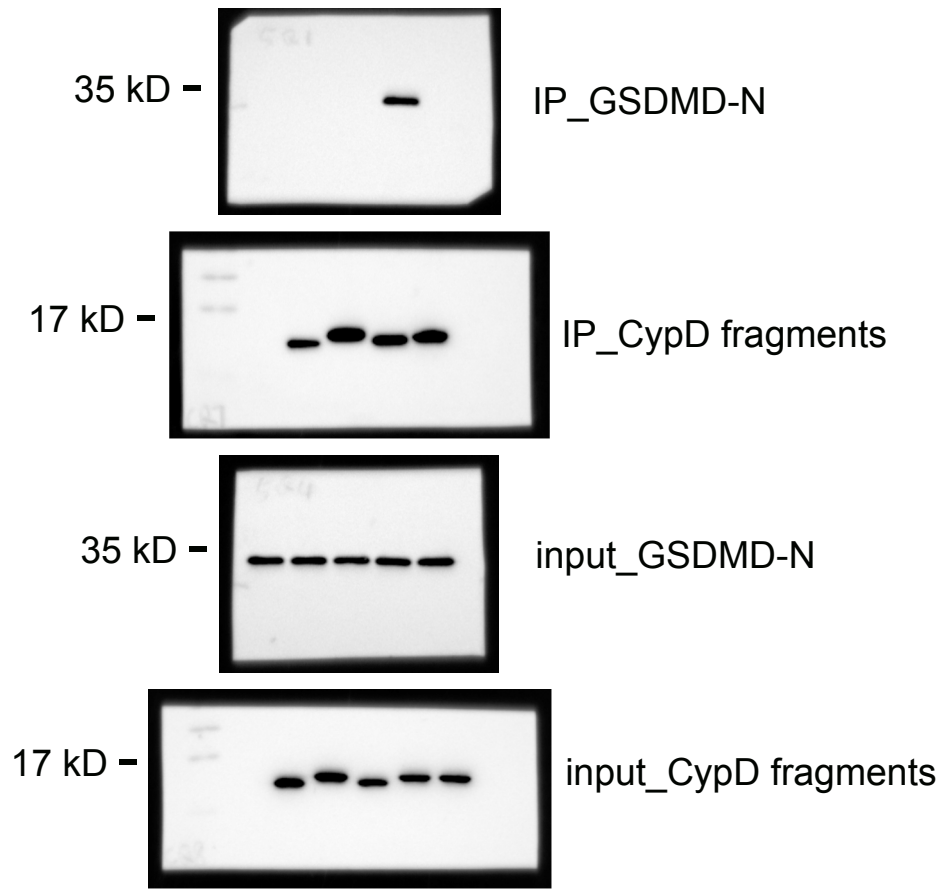

Fig.S6F

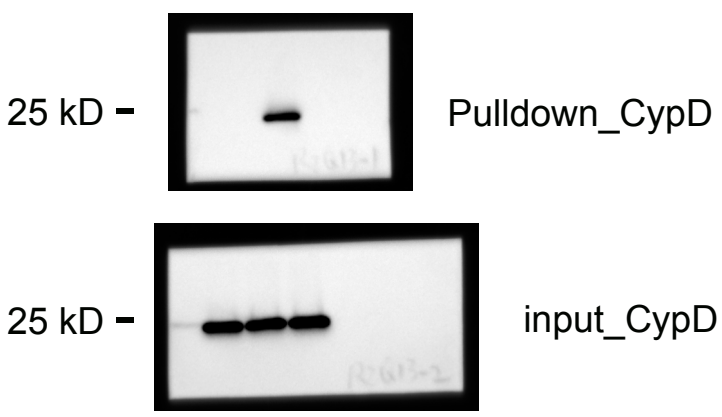

Supplement: Supplementary file 1 [file ppat.1014554.s009.pdf]
